# Activation of transmembrane receptor tyrosine kinase DDR1-STAT3 cascade by extracellular matrix remodeling promotes liver metastatic colonization in uveal melanoma

**DOI:** 10.1038/s41392-021-00563-x

**Published:** 2021-05-12

**Authors:** Wei Dai, Shenglan Liu, Shubo Wang, Li Zhao, Xiao Yang, Jingfeng Zhou, Yun Wang, Jing Zhang, Ping Zhang, Ke Ding, Yangqiu Li, Jingxuan Pan

**Affiliations:** 1 Jinan University Institute of Tumor Pharmacology, College of Pharmacy, Jinan University, Guangzhou, China; 2 Integrated Chinese and Western Medicine Postdoctoral Research Station, Jinan University, Guangzhou, China; 3 State Key Laboratory of Ophthalmology, Zhongshan Ophthalmic Center, Sun Yat-sen University, Guangzhou, China; 4 International Cooperative Laboratory of Traditional Chinese Medicine Modernization and Innovative Drug Development of Chinese Ministry of Education (MOE), Guangzhou City Key Laboratory of Precision Chemical Drug Development, College of Pharmacy, Jinan University, Guangzhou, China

**Keywords:** Metastasis, Eye cancer, Target validation

## Abstract

Colonization is believed a rate-limiting step of metastasis cascade. However, its underlying mechanism is not well understood. Uveal melanoma (UM), which is featured with single organ liver metastasis, may provide a simplified model for realizing the complicated colonization process. Because DDR1 was identified to be overexpressed in UM cell lines and specimens, and abundant pathological deposition of extracellular matrix collagen, a type of DDR1 ligand, was noted in the microenvironment of liver in metastatic patients with UM, we postulated the hypothesis that DDR1 and its ligand might ignite the interaction between UM cells and their surrounding niche of liver thereby conferring strengthened survival, proliferation, stemness and eventually promoting metastatic colonization in liver. We tested this hypothesis and found that DDR1 promoted these malignant cellular phenotypes and facilitated metastatic colonization of UM in liver. Mechanistically, UM cells secreted TGF-β1 which induced quiescent hepatic stellate cells (qHSCs) into activated HSCs (aHSCs) which secreted collagen type I. Such a remodeling of extracellular matrix, in turn, activated DDR1, strengthening survival through upregulating STAT3-dependent Mcl-1 expression, enhancing stemness via upregulating STAT3-dependent SOX2, and promoting clonogenicity in cancer cells. Targeting DDR1 by using 7rh, a specific inhibitor, repressed proliferation and survival in vitro and in vivo outgrowth. More importantly, targeting cancer cells by pharmacological inactivation of DDR1 or targeting microenvironmental TGF-β1-collagen I loop exhibited a prominent anti-metastasis effect in mice. In conclusion, targeting DDR1 signaling and TGF-β signaling may be a novel approach to diminish hepatic metastasis in UM.

## Introduction

Distant organ metastasis (in particular to life-maintaining organs such as liver, brain, and lung) is one of the important causes of therapeutic failure and mortality in patients with cancers at the terminal stage. While patients with breast cancer, lung cancer, cutaneous melanoma manifest metastasis to different organ sites,^1^ patients with uveal melanoma (UM) are featured with single organ liver metastasis.^2^ The single organ-specific metastasis may provide a simplified model for realizing the complicated metastasis process.

Metastasis can be principally dissected into intravasation, circulation, extravasation, and colonization. Colonization is a rate-limiting step of metastasis cascade. It was reported that in a melanoma experimental metastasis model, the majority (>80%) of injected tumor cells can survive the circulation and successfully extravasate into liver. Despite so, only 1 out of 40 cells formed micrometastases by day 3, and only 1 out of 100 micrometastases grew into macroscopic metastases by day 10.^3^

Colonization is believed an outcome of the interaction between “seed and soil” through cell–cell and cell–extracellular matrix (ECM) adhesion and the release of soluble factors, which eventually allow cancer cells to gain strengthened survival capacity, strengthened proliferative capacity, strengthened stemness, and construction of supportive niche.^4^ Drivers of colonization are not well understood. However, during such a colonization process, ECM is postulated to not only serve a scaffold of outgrowth, but also provide an amplifier of oncogenic signaling in tumor cells because of its unique features of three-dimensional super molecular structures with distinct biochemical and biomechanical properties that regulate cellular survival, proliferation and stemness by ligating specific receptors.^5^ Collagen deposition, elevated expression of matrix remodeling genes such as MMPs and collagen cross-linkers are observed to be predictive of a poor prognosis in patients with breast cancer and pancreatic cancer.^6^ Patients with fibrosis and cirrhosis of organs such as liver and lung in which abnormal accumulation of collagen have an increased risk of metastasis in pancreatic ductal adenocarcinoma (PDAC).^7^

The increased collagen deposition can ligate their corresponding surface receptors [e.g., the discoidin domain receptor (DDR) family of receptor tyrosine kinases and certain types of integrin (e.g., α1β1, α2β1, α10β1, and α11β1)] of cancer cells.^8^ DDR, composed of two members, DDR1 and DDR2, regulates downstream molecules (e.g., STAT3, STAT5),^9^ which eventually coordinates cell adhesion, migration, proliferation, and matrix remodeling.^9^

Given that the microenvironment of liver in metastatic patients with UM contains pathological collagen deposition,^10^ we hypothesized that DDR and its ligand might ignite the interaction between metastasis-initiating cells and their surrounding niche thereby conferring colonization. In the present study, we tested this hypothesis by dissecting the expression of DDR1 and its signaling in UM cells. We found that DDR1-overexpressing UM cells educated hepatic stellate cells (HSCs) to secret collagen I, which in turn ligated DDR1 to activate UM cells to release HSCs-activating soluble cytokine TGF-β1, forming a positive loop to enhance survival, proliferation and stemness, which eventually promotes liver metastasis. These findings may shed light on the understanding of modus operandi by which tumor cells seduce the host liver microenvironment, providing rationale targets for liver metastatic colonization in UM.

## Results

### DDR1 is overexpressed in cell lines and primary specimens of UM

**Fig. 1:**
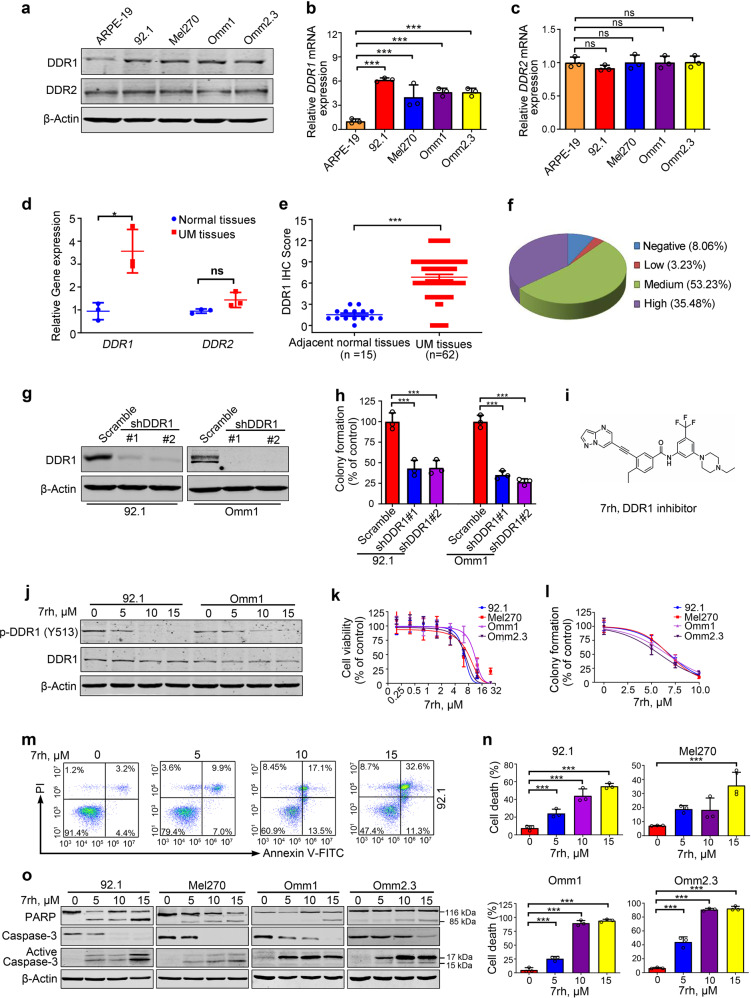
DDR1 is overexpressed in uveal melanoma and inhibition of DDR1 reduces colony formation capacity and induces apoptosis in human UM cells. **a**–**c** Protein and mRNA levels of DDR1 and DDR2 in human uveal melanoma (UM) cells (Mel270, 92.1, Omm1, and Omm2.3) and retinal pigment epithelial (ARPE-19) cells were determined by western blotting and qRT-PCR analysis, respectively. ns, no significant. Data are shown as the mean ± SD (*n* = 3). **d** The mRNA levels of *DDR1* and *DDR2* genes in normal choroid tissues and UM tissues were assessed by qRT-PCR. Data are shown as the mean ± SD (*n* = 3). **e**, **f** Statistical analysis of DDR1 expression in paraffin-embedded tissues from the patients with UM (*n* = 62) is shown. The staining intensity was scored on four levels (negative, low, medium, and high). Data are shown as the mean ± SD. **g** 92.1 and Omm1 cells stably transduced with control shRNA (Scramble) or two DDR1 shRNAs lentivirus underwent western blotting analysis or **h** colony-formation assay in soft agar-containing culture, Data are shown as the mean ± SD (*n* = 3). **i** The chemical structure of DDR1 inhibitor 7rh is shown. **j** The effect of 7rh on the phosphorylation of DDR1 was ascertained by western blotting analysis after 92.1 and Omm1 cells exposure to 7rh for 24 h. **k** UM cells were treated with escalating concentrations of 7rh for 72 h, cell viability was determined by MTS assay. Data are shown as the mean ± SD (*n* = 3). **l** After treatment with 7rh at the indicated concentrations for 48 h, the UM cells were harvested and seeded in drug-free soft agar-containing culture for 14 days, colonies were counted and analyzed. Data are shown as the mean ± SD (*n* = 3). **m**, **n** UM cells were exposed to increasing concentrations of 7rh for 48 h, or 15 μM 7rh for the different durations, flow cytometry was employed to detect the apoptosis after dual-staining with Annexin V-FITC and PI. Representative flow cytometry dot plots for 92.1 cells (**m**) and quantitative analysis of results from three independent experiments (**n**) are shown. The *Y*-axis is the sum of the top left, top right, and bottom right quadrants. Data are shown as the mean ± SD (*n* = 3). **o** Cleavage of PARP and activation of caspase-3 were determined by western blotting after UM cells were treated with escalating concentrations of 7rh. *P < 0.05; ***P < 0.001, one-way ANOVA, post hoc comparisons, Tukey’s test for result in **b**, **c**, **h** and **n**; *P < 0.05; ***P < 0.001, Student’s *t* test for results in (**d**, **e**)

Western blotting analysis showed that the protein levels of DDR1 rather than DDR2 were considerably higher in the four lines of human UM cells than those in ARPE-19 cells (Fig. 1a). In parallel, qRT-PCR analysis revealed that the mRNA levels of *DDR1* rather than *DDR2* were significantly increased in the UM cells relative to ARPE-19 cells (Fig. 1b, c). The significantly increased mRNA levels of *DDR1* were also found in the primary UM tissues versus normal choroid tissues (Fig. 1d). These results suggest that the overexpression of DDR1 in UM cells occurs at the transcriptional layer. After the specificity of the anti-DDR1 antibody was verified (Supplementary Fig. S1a), we measured the expression of DDR1 in the primary ocular tumor specimens from patients with UM by using immunohistochemistry (IHC) staining with anti-DDR1. In contrast to the undetectable levels of DDR1 in the adjacent normal tissues (Supplementary Fig. S1b), the positive staining (ranging from low to high by IHC score) of DDR1 was observed in 57 out of 62 (92%) of the tested UM cases (Fig. 1e, f). Moreover, the expression of DDR1 was positively correlated with the largest basal diameter (*P* = 0.00006) and thickness (*P* = 0.0386) of primary tumors, which are two important independent predictors of UM metastatic death.^11^ It was also positively correlated with the TNM stage (*P* = 0.0019) in cohort (Supplementary Table S2). Taken together, these results suggest that the overexpressed DDR1 predicts a poor prognosis in UM.

### DDR1 promotes cellular proliferation in UM cells

The overexpression of DDR1 in UM cells and tissues prompted us to ask whether DDR1 promoted the growth of UM cells. 92.1 and Omm1 cells with DDR1 stably silenced by lentiviral shRNA manifested a significantly decreased clonogenicity as evaluated in soft agar-containing culture (Fig. 1g, h). We next employed 7rh (Fig. 1i), a specific small-molecule inhibitor of DDR1 (15-fold selectivity relative to DDR2) as described in our previous report,^12^ to confirm the role of DDR1 in UM cells. UM cells were treated with increasing concentrations of 7rh for 48 h, western blotting analysis showed that 7rh decreased the phosphorylation of DDR1 without alternating the protein levels of DDR1, suggesting that 7rh can effectively inhibit the cellular DDR1 kinase activity in UM cells (Fig. 1j). Cell viability assay showed that 7rh concentration-dependently dampened the growth of UM cells with IC_50_ values ranged from 4 to 10 μM (Fig. 1k). Separately, the UM cells were treated with increasing concentrations of 7rh for 24 h, and then seeded in soft agar culture in the absence of 7rh. The colony formation was concentration-dependently reduced by 7rh (Fig. 1l). Taken together, these data suggest that DDR1 promotes the proliferation of UM cells.

### DDR1 enhances cellular survival via pro-survival Mcl-1 gene

Because cellular survival is a critical prerequisite for colonization when cancer cells are disseminated into host organs, the pro-survival role of DDR1 in UM cells was examined. The UM cells were treated with various concentrations of 7rh for 48 h or a fixed concentration (15 µM) for various durations, apoptosis was assessed by flow cytometry after dually stained with Annexin V-FITC and propidium iodide (Fig. 1m). The results showed that percentages of dead cells were increased in a concentration- and time-dependent manner after 7rh treatment (Fig. 1n and Supplementary Fig. S2a). As well, 7rh induced a concentration- (Fig. 1o) and time-dependent (Supplementary Fig. S2b) PARP cleavage and caspase-3 activation. 7rh treatment elicited an increase in cell population with loss of mitochondrial potential (ΔΨm), as measured by flow cytometry after chloromethyl-X-rosamine and MTGreen double staining in UM cells (Supplementary Fig. S2c, d). Collectively, these data together suggest that 7rh may induce mitochondrial damage and apoptosis in UM cells.

### 7rh downregulates Mcl-1 and induces apoptosis in UM cells

To investigate the mechanism of 7rh-induced apoptosis in UM cells, we evaluated the expression of apoptosis-related proteins. Western blotting analysis showed that the protein levels of pro-survival Mcl-1 were obviously decreased without considerably change in the levels of other apoptosis-related family members (XIAP, Bcl-2, Bcl-X_L_, Bax, and Survivin) (Supplementary Fig. S3a). We next determined the role of Mcl-1 in 7rh-induced apoptotic cell death in UM cells. Forced overexpression of Mcl-1 attenuated the 7rh-induced apoptosis in Mel270 cells as reflected by PARP cleavage (Supplementary Fig. S3b, left) and trypan blue staining cells (Supplementary Fig. S3c). Conversely, knockdown of Mcl-1 by siRNA duplexes significantly accelerated the lethal effect of 7rh in UM cells (Supplementary Fig. S3b, right and Supplementary Fig. S3d). These results reveal that DDR1 may exert its pro-survival role via Mcl-1.

### 7rh downregulates Mcl-1 transcription through STAT3

We further explored the underlying mechanism that Mcl-1 was downregulated by DDR1 inhibition. qRT-PCR analysis showed that DDR1 deletion inhibited the transcription of Mcl-1 in UM cells (Supplementary Fig. S3e). It has been reported that Mcl-1 is the target gene of transcription factor STAT3 implicated in tumor survival.^13^ In addition, STAT3 is a critical downstream of DDR1 signaling,^14^ we, therefore, hypothesized that STAT3 may be a mediator between DDR1 and Mcl-1 in UM. As expected, ChIP assay indicated that knockdown of DDR1 obviously decreased the STAT3 binding to the promoter of Mcl-1 gene, which was reversed by forced overexpressing STAT3 in Mel270 cells. There was no significant enrichment at a downstream intronic region on Mcl-1 lacking STAT3 binding sequence (Supplementary Fig. S3f, g).

### Pharmacological inactivation of DDR1 tyrosine kinase impedes outgrowth of xenografted Omm1 cells and PDX in NOD-SCID mice

**Fig. 2:**
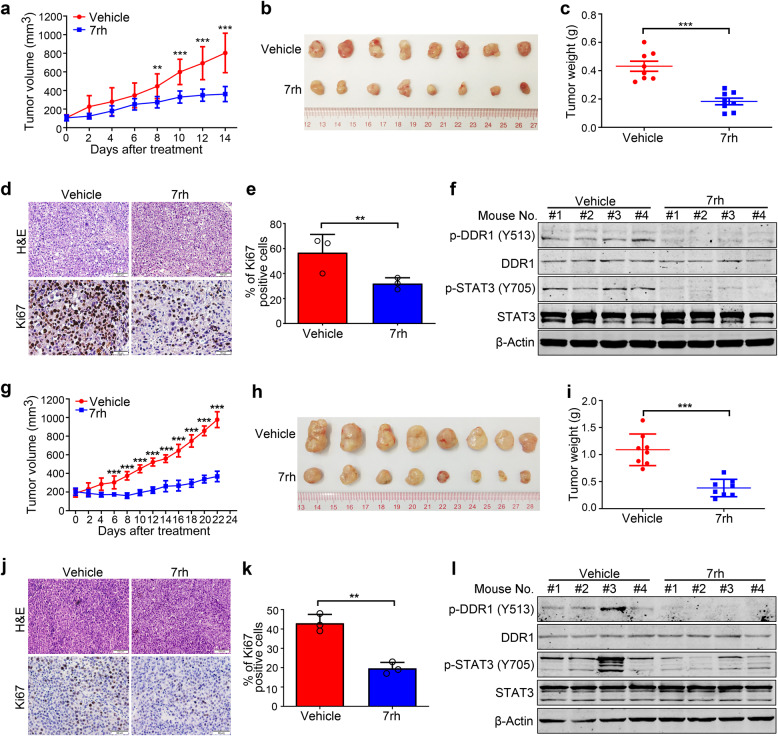
7rh impedes outgrowth of xenografted Omm1 cells and MP41 cells patient-derived xenograft model in NOD-SCID mice. **a**–**c** Male NOD-SCID mice (4–6 weeks) bearing palpable Omm1 xenografted tumors were randomly administered with either vehicle (ddH_2_O:DMSO:EtOH:Cremophor EL = 90:2:4:4) or 7rh (25 mg/kg in vehicle, orally) every day for 14 days (*n* = 8 per group). **a** The tumor volumes estimated every other day measurement by caliper versus time were plotted. Data are shown as the mean ± SD (*n* = 8). **b** Representative tumors resected from mice treatment with vehicle or 7rh for 14 days are shown. **c** Weights of tumor from mice of each group are shown. Data are shown as the mean ± SD (*n* = 8). **d** The xenograft tissues from the vehicle- or 7rh-treated mice were subject to H&E staining and IHC analysis with anti-Ki67. Scale bar: 100 µm (H&E staining), 50 µm (IHC staining). **e** The total number of Ki67-positive cells (brown-stained nuclei, regardless of staining intensity were counted as positive) in three random microscopic fields was counted by Image-Pro Plus 6.0. Data are shown as the mean ± SD (*n* = 3). **f** Western blotting detection of DDR1, phospho-DDR1 and its downstream signal phospho-STAT3 in tumor tissues from each group of mice is shown. **g**–**l** The effect of 7rh on UM PDX model. Male NOD-SCID mice (4–6 weeks) bearing palpable MP41 cells xenografted tumors were randomly administered with either vehicle (ddH2O:DMSO:EtOH:Cremophor EL = 90:2:4:4) or 7rh (25 mg/kg in vehicle, orally) every day for 22 days. **g** The tumor volumes estimated every other day measurement by caliper versus time were plotted. Data are shown as the mean ± SD (*n* = 8). **h**, **i** The mice were sacrificed and tumors were collected, then tumors were photographed and weights were measured and analyzed. Data are shown as the mean ± SD (*n* = 8). **j**, **k** Tumor sections from two groups were subject to H&E staining and IHC analysis with anti-Ki67. The total number of Ki67-positive cells was counted. Data are shown as the mean ± SD (*n* = 3). Scale bar: 100 µm (H&E staining), 50 µm (IHC staining). **l** Western blotting analysis of cell lysates of tumors was performed to detect DDR1, phospho-DDR1 and its downstream signal phospho-STAT3 after vehicle or 7rh treatment. **P < 0.01; ***P < 0.001, Student’s *t* test for results in (**a**, **c**, **e**, **g**, **i** and **k**)

To evaluate the in vivo function of DDR1 activity, the NOD-SCID mice (4–6-week-old) were subcutaneously injected with Omm1 cells. When the tumor xenografts were palpable (~100 mm^3^), the mice were randomly divided into two groups administered with vehicle or 7rh for 2 weeks. The results showed that the increase in tumor volume was impeded in 7rh-treated group when compared to vehicle-treated mice (Fig. 2a). Consistently, tumor weight of the 7rh-treated mice was significantly lower than that of the vehicle-treated mice (Fig. 2b, c). Furthermore, IHC staining assessment of Ki67 validated the remarkable decrease of cell proliferation upon 7rh treatment (Fig. 2d, e). Western blotting analysis of cell lysates from four tumors of each group indicated that the phosphorylation of DDR1 and its downstream signaling STAT3 were blocked after 7rh administering (Fig. 2f). Similar results were obtained in a human UM MP41 PDX model (Fig. 2g–l). These data demonstrate that pharmacological inactivation of DDR1 kinase by 7rh impedes the outgrowth of UM cells in vivo.

### DDR1 confers maintenance of cancer stem-like cell traits via SOX2 in UM

**Fig. 3:**
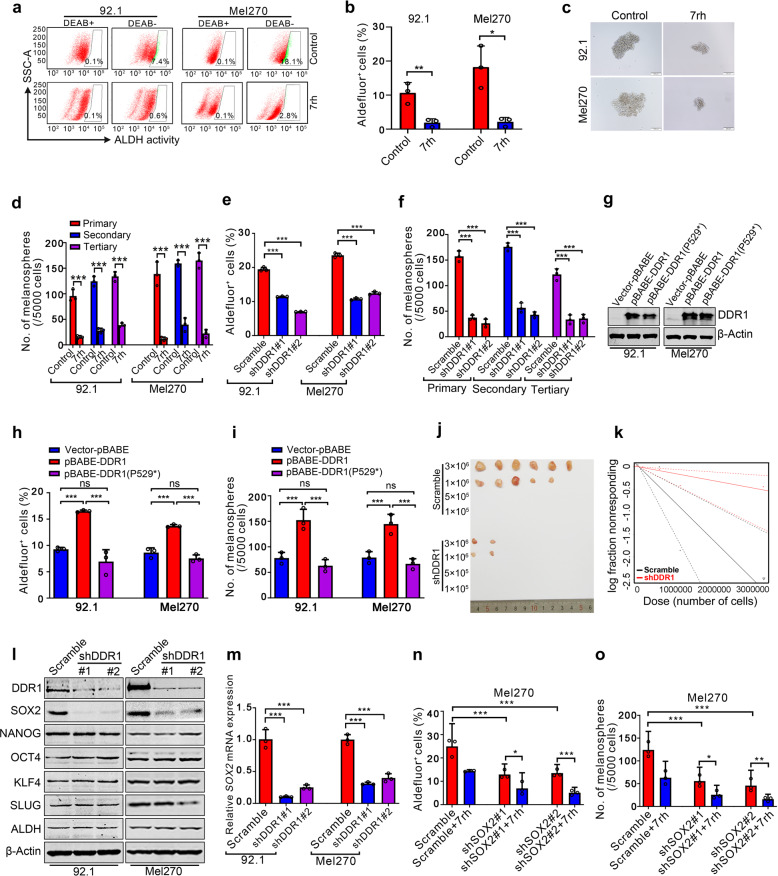
DDR1 inhibition suppresses the properties of cancer stem-like cells through lowering SOX2 in UM cells. **a, b** 92.1 and Mel270 cells were treated with 10 μM 7rh for 48 h, the ALDH activity was detected by flow cytometry. Representative flow cytometry (**a**) and quantitative analysis of ALDH^+^ cells (**b**) from three independent experiments are shown. Data are shown as the mean ± SD (*n* = 3). **c**, **d** 92.1 and Mel270 cells were treated with 10 μM 7rh for 48 h, and then drug-freely cultured for three rounds of melanosphere assay for 14 days. Data are shown as the mean ± SD (*n* = 3). Scale bar: 200 µm. **e** 92.1 and Mel270 cells stably transduced with lentiviral Scramble or shDDR1 were assayed by flow cytometry for the proportion of ALDH^+^ cells. Quantitative analysis of ALDH^+^ cells from three independent experiments is shown. Data are shown as the mean ± SD (*n* = 3). **f** 92.1 cells stably transduced with lentiviral Scramble or shDDR1 were plated in the stem cell culture medium. Melanospheres were counted on day 14. The cells were harvested and replated for the secondary and tertiary rounds of evaluation, respectively. Data are shown as the mean ± SD (*n* = 3). ***P < 0.001, one-way ANOVA, post hoc comparisons, Tukey’s test. **g** Ectopic expression of wild-type DDR1 or mutant DDR1 (DDR1 P529*) in 92.1 and Mel270 cells were determined by western blotting analysis. **h** Overexpression of wild-type DDR1 but not mutant DDR1 (DDR1 P529*) increased the percentage of ALDH^+^ cells as detected by flow cytometry in 92.1 and Mel270 cells. ns no significant. Data are shown as the mean ± SD (*n* = 3). **i** Ectopic expression of wild-type DDR1 rather than mutant DDR1 (DDR1 P529*) potentiated self-renewal capacity as evaluated by melanosphere growth and serially-replating assay in 92.1 and Mel270 cells. ns no significant. Data are shown as the mean ± SD (*n* = 3). **j**, **k** Silencing DDR1 decreased in vivo frequency of CSCs in UM cells. Omm1 cells stably transduced with lentiviral Scramble or shDDR1 were subjected to limiting dilution assay in NOD-SCID mice. Representative image of tumors removed from the mice of each group (*n* = 6) are shown. The frequency of CSCs was calculated by L-Cal software. **l** DDR1 deletion decreased the expression of SOX2. The protein levels of stemness-related proteins were detected by western blotting in 92.1 and Mel270 cells stably transduced with Scramble or shDDR1 lentivirus. **m** DDR1 deletion downregulated the mRNA levels of SOX2. The mRNA levels of SOX2 in 92.1 and Mel270 cells stably transduced with Scramble or shDDR lentivirus were determined by qRT-PCR. Data are shown as the mean ± SD (*n* = 3). **n**, **o** The percentage of ALDH^+^ cells and melanosphere assay were performed in Mel270 cells stably transduced with Scramble or shSOX2 lentivirus with or without 7rh. Data are shown as the mean ± SD (*n* = 3). *P < 0.05; **P < 0.01; ***P < 0.001, Student’s *t* test for results in (**b**, **d)**. *P < 0.05; **P < 0.01; ***P < 0.001, one-way ANOVA, post hoc comparisons, Tukey’s test for results in (**e**, **f**, **h**, **i** and **m–o**)

Because cancer stem-like cells (CSCs) disseminated into the host organs likely function as ‘seeds’ of colonization,^15^ we asked whether DDR1 maintained the CSC traits. Exposure of UM cells to 7rh to block DDR1 kinase activity resulted in a significant decrease of the percentage of ALDH^+^ cells (Fig. 3a, b and Supplementary Fig. S4a). 7rh also abrogated the self-renewal capacity as reflected by the serially-replating melanosphere ability (Fig. 3c, d and Supplementary Fig. S4b). Consistently, silencing DDR1 by lentiviral shRNA against DDR1 significantly eliminated CSC properties as displayed repressed the percentage of ALDH^+^ cells (Fig. 3e and Supplementary Fig. S4c) and serial melanosphere formation (Fig. 3f and Supplementary Fig. S4d). While forced expression of wild-type DDR1 but not the loss-of-function mutant DDR1 (Fig. 3g and Supplementary Fig. S4e) significantly elevated the percentage of ALDH^+^ cells (Fig. 3h and Supplementary Fig. S4f) as well as serial melanosphere formation (Fig. 3i and Supplementary Fig. S4g). Furthermore, in vivo limiting dilution assay revealed that silencing DDR1 by shRNA reduced UM CSCs frequency 5.3-fold (Scramble: 1.09 × 10^−6^; shDDR1: 5.82 × 10^−6^) (Fig. 3j, k and Supplementary Table S3). All these data indicate that abolishing DDR1 eliminates CSCs in UM.

To explore the molecular mechanism that DDR1 maintains the phenotypes of CSCs, we detected the expression of stemness-associated proteins. Western blotting assay revealed an appreciable decrease in SOX2 in DDR1-silenced UM cells without obvious alteration in SLUG, KLF4, NANOG, ALDH, and OCT4 (Fig. 3l and Supplementary Fig. S4h). qRT-PCR analysis showed that the mRNA levels of *SOX2* were also inhibited in DDR1-silenced cells (Fig. 3m). Notably, disrupting SOX2 by shRNA diminished the percentage of ALDH^+^ cells and serially-replating capacity of melanosphere formation (Fig. 3n, o). Silencing SOX2 potentiated the 7rh-mediated decrease in ALDH^+^ cells and melanosphere formation in UM cells (Fig. 3n, o). Collectively, these results indicate that SOX2 positively regulates CSCs properties, and that DDR1 inactivation by 7rh decreases SOX2 and eradicates CSCs in UM cells.

### DDR1 knockdown diminishes liver metastatic colonization in UM

**Fig. 4:**
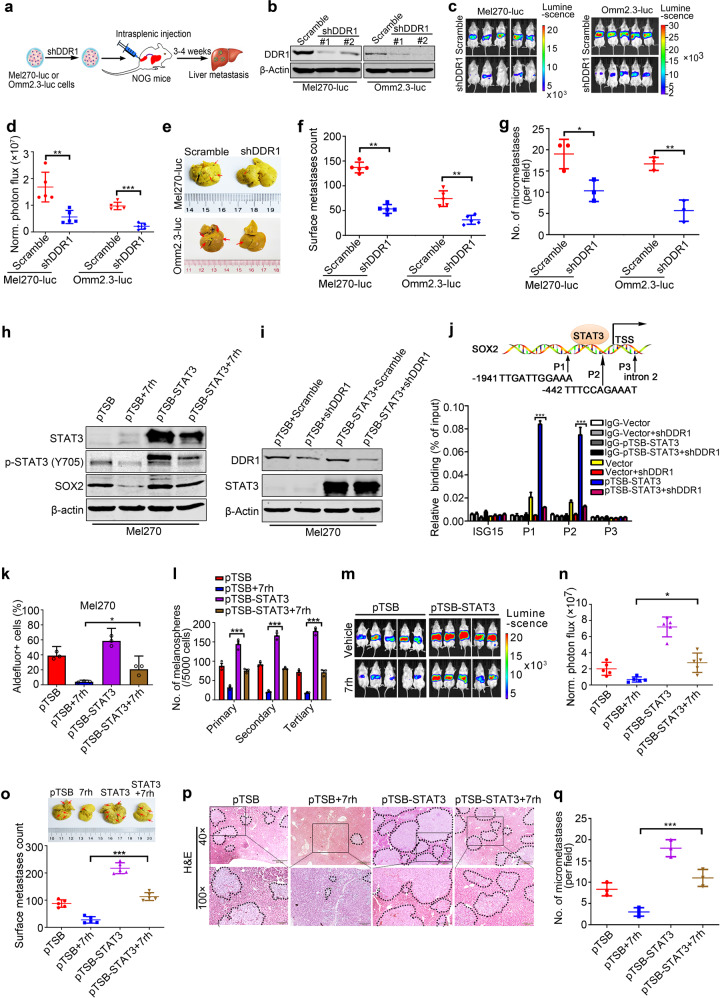
Silencing DDR1 suppresses the metastatic colonization of UM cells in liver. **a** Experimental schematic of liver metastasis model. **b** DDR1 knockdown in Mel270-luc and Omm2.3-luc cells stably transduced with Scramble or shDDR1 lentivirus was confirmed by Western blotting. **c, d** Mel270-luc and Omm2.3-luc cells stably transduced with Scramble or shDDR1 were intrasplenically inoculated in NOG mice for 3–4 weeks (*n* = 5 per group). Liver metastasis was analyzed by luciferase-based bioluminescence imaging system. Representative images (**c**) and quantitative analysis (**d**) of photon flux on day 21 are shown. Data are shown as the mean ± SD (*n* = 5). **e**, **f** The mice were sacrificed to count metastatic nodules on liver surface. Representative images (**e**) and quantitative analysis of liver surface nodules (**f**) are shown. Data are shown as the mean ± SD (*n* = 5). **g** Quantitative analysis of micrometastases in H&E staining liver sections from Scramble and shDDR1 mice. Data are shown as the mean ± SD (*n* = 3). **P < 0.01, Student’s *t* test for results in (**d**, **f**, **g**). **h** Mel270 cells transduced with lentiviral vector (pTSB) or construct encoding human STAT3 cDNA (pTSB-STAT3) were exposed to 10 μM 7rh for 24 h, and subjected to western blotting analysis with the indicated antibodies. **i** DDR1 knockdown and STAT3 overexpression was confirmed by western blotting in Mel270 cells stably expressed shDDR1 with or without STAT3 overexpression. **j** Experimental schematic diagram showing the location of STAT3-binding sites of SOX2 regulatory region. P1, P2 represent STAT3-binding sites at the SOX2 gene promoter. P3 was used as negative control and located in intronic region. ISG15 as a known non-target gene of STAT3 served as a negative control (top). ChIP-PCR analysis for STAT3 occupancy at the SOX2 gene promoter in Mel270 cells stably expressed shDDR1 with or without STAT3 overexpression (bottom). Data are shown as the mean ± SD (*n* = 3). **k** Enforced expression of STAT3 attenuated the 7rh-mediated decrease in percentage of ALDH^+^ cells in UM cells. Mel270 cells stably expressed with STAT3 were treated with or without 10 μM 7rh for 24 h, and then subjected to ALDH^+^ cells analysis by flow cytometry. Quantitive analysis of ALDH^+^ cells from three independent experiments is shown. Data are shown as the mean ± SD (*n* = 3). **l** Overexpression of STAT3 reversed the 7rh-mediated decrease in melanosphere growth and serially-replating capacity in UM. Mel270 cells stably expressed with STAT3 were treated with or without 10 μM 7rh for 24 h, and then subjected to melanosphere-formation assay. Data are shown as the mean ± SD (*n* = 3). **m**–**q** Mel270-luc cells stably transduced with lentiviral vector (pTSB) or constructs encoding human STAT3 cDNA (pTSB-STAT3) underwent intrasplenic injection in NOG mice, the mice were then administrated with vehicle (ddH2O:DMSO:EtOH:Cremophor EL = 90:2:4:4) or 7rh (25 mg/kg in vehicle, orally) every day for 21 days (*n* = 5 per group). Liver metastasis was analyzed by luciferase-based bioluminescence imaging and liver surface nodules were counted. Representative images were taken on day 21 post injection of cells (**m**) and quantitative analysis of bioluminescence intensity (**n**) are shown. Data are shown as the mean ± SD (*n* = 5). **o** Representative bright field images of liver surface and quantitative analysis of surface metastatic nodules on liver are shown. Data are shown as the mean ± SD (*n* = 5). **p**, **q** Microscopic observation of H&E staining in liver paraffin section from each group verified metastastic nodules. Data are shown as the mean ± SD (*n* = 3). Scale bar: 500 µm (40×), 200 µm (100×). *P < 0.05; ***P < 0.001, one-way ANOVA, post hoc comparisons, Tukey’s test for results in (**j**, **k**, **l**, **n**, **o** and **q**)

To assess the effect of DDR1 on metastatic colonization, Mel270-luc or Omm 2.3-luc cells stably transduced with lentiviral Scramble or shDDR1 were intrasplenically injected into NOG mice (Fig. 4a). We found that DDR1 knockdown (Fig. 4b) effectively retarded bioluminescence signals in livers on day 21 (Fig. 4c, d), as well as restrained the counts of liver surface metastatic nodules (Fig. 4e, f). In accordance, H&E staining showed a remarkable reduction in density and size of the metastatic nodules in the livers of shDDR1 group relative to those in the Scramble group (Fig. 4g and Supplementary Fig. S5a, b). These results demonstrate that DDR1 inhibition suppresses metastatic colonization in UM.

### STAT3-dependent SOX2 upregulation by DDR1 facilitates traits of CSCs and metastasis in UM

Given that STAT3 can directly bind to the promoter of SOX2 gene and that DDR1 can activate STAT3,^14,16^ we detected whether DDR1 mediated SOX2 transcription via STAT3 in UM. Mel270 cells transduced with lentiviral STAT3-encoding construct were exposed to 7rh for 24 h, western blotting analysis and CSCs traits were evaluated. Western blotting analysis unveiled that the forced expression of STAT3 reversed the 7rh-mediated decrease in SOX2 (Fig. 4h). The results of ChIP assay revealed that depletion of DDR1 attenuated the abundance of STAT3 at the promoter region of *SOX2* compared with Scramble control cells, which was at least partially reversed by forced expression of STAT3 (Fig. 4i, j). There was no significant enrichment at a downstream intronic region on *SOX2* lacking STAT3 binding sequence. These results indicate that STAT3 is fundamental for DDR1-mediated SOX2 transcription.

Moreover, forced expression of STAT3 elevated the proportion of ALDH^+^ cells (Fig. 4k) as well as serial melanosphere formation (Fig. 4l). Ectopic expression of STAT3 attenuated the 7rh-mediated decrease in the percentage of ALDH^+^ cells (Fig. 4k) and melanoshpere formation and replating ability (Fig. 4l). We next carried out experiments to validate the effect of STAT3 on metastatic colonization. As expected, administration of 7rh significantly decreased the bioluminescence signal intensity of liver tumor burdens (Fig. 4m, n), as well as the number of metastatic nodules on liver surface (Fig. 4o–q), which was at least partially reversed by forced expression of STAT3. Taken together, these data suggest that STAT3 is required for DDR1 to promote CSCs, and metastatic colonization in UM.

### UM cells educate hepatic stellate cells to secret type Ia collagen

**Fig. 5:**
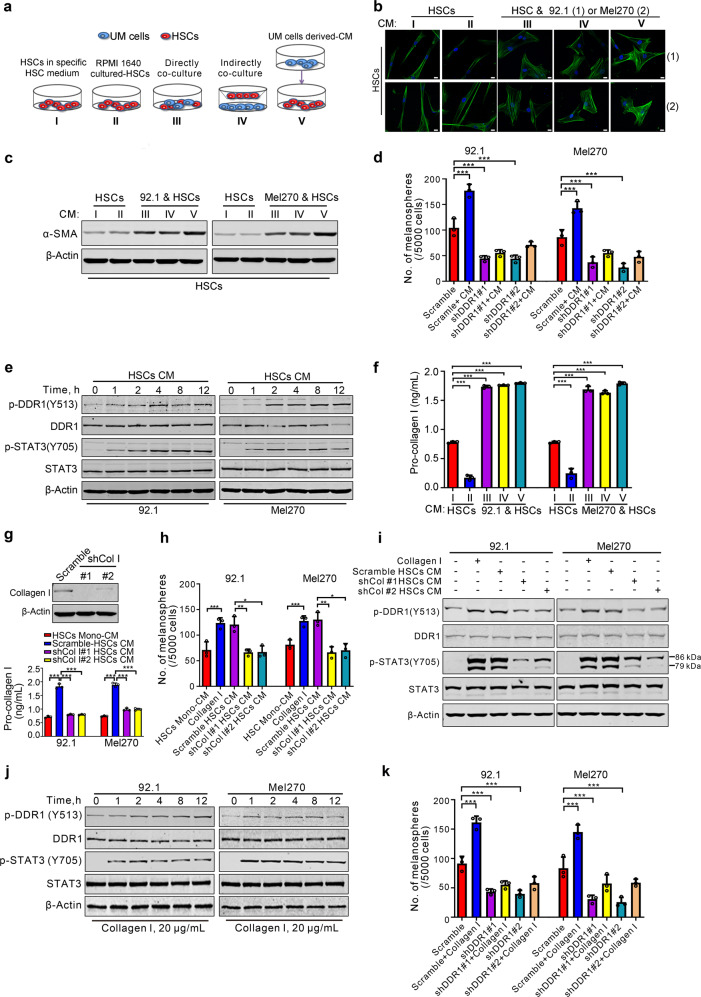
UM cells educate hepatic stellate cells (HSCs) to secret collagen I. **a** Schema of mono-cultured HSCs or co-cultured HSCs with UM cells. I, mono-cultured HSCs with HSC-specific culture medium; II, mono-cultured HSCs with RPMI 1640 culture medium; III, directly co-culture HSCs mixed 1:1 with UM cells for 24 h; IV, HSCs were co-cultured with UM cells in Transwell systems for 24 h; V, UM cells-derived conditioned medium (CM) were added to culture starved HSCs for 6 h and replaced with HSCs specific medium for another 24 h. **b**, **c** CM were isolated from HSCs grown in mono-culture or co-cultured with 92.1 or Mel270 cells as indicated approaches (**a**), and then added into the culture of HSCs for 24 h, the marker of activated-HSCs α-SMA was determined by immunofluorescence assay (**b**) and western blotting (**c**). Scale bar: 10 µm. **d** DDR1 is required for melanosphere-formation in UM cells stimulated by HSCs-derived CM. 92.1 and Mel270 cells stably expressing Scramble or shDDR1 were cultured with HSCs-derived CM and then subjected to melanosphere assay. The medium of UM cells was added to culture starved HSCs for 6 h and replaced with HSCs specific medium for another 24 h, which collected as HSCs-derived CM. Data are shown as the mean ± SD (*n* = 3). **e** The CM from HSCs that were educated by UM cells activated DDR1 kinase in UM cells. The HSCs-derived CM was added into the culture of 92.1 and Mel270 cells for different times, and then phosphorylation of DDR1 and its downstream signal phosphorylation of STAT3 were analyzed by western blotting. **f** ELISA evaluation of the collagen type Iα was performed in various CM collected from different culture systems as illustrated in (**a**). Data are shown as the mean ± SD (*n* = 3). **g** HSCs were infected with lentiviral Scramble or specific shRNA targeting collagen type I, and verified by western blotting (top). The level of collagen type Iα in the CM harvested from HSCs that were stably transduced with lentiviral Scramble or shCol I were measured by ELISA assay (bottom). Data are shown as the mean ± SD (*n* = 3). **h** The medium of 92.1 or Mel270 cells was added to culture starved HSCs expressing Scramble or two shRNAs against Collagen I for 6 h and replaced with HSCs specific medium for another 24 h, which collected as Scramble HSC CM, shCol I #1 HSC CM, and shCol I #2 HSC CM, respectively. The indicated HSC-derived CM and recombinant collagen type I (10 μg/mL) were used for melanosphere assay in 92.1 or Mel270 cells. Data are shown as the mean ± SD (*n* = 3). **i** Western blotting was conducted after 92.1 or Mel270 cells incubation with recombinant collagen type I (20 μg/mL) or Scramble HSC CM, shCol I #1 HSC CM, and shCol I #2 HSC CM as illustrated in (**h**) for 24 h. **j** After UM cells treated with 20 μg/mL collagen I for different durations, the phosphorylation of DDR1 and STAT3 were examined by western blotting. **k** 92.1 and Mel270 cells stably transduced lentiviral Scramble or shDDR1 were cultured in the absence or presence of recombinant human collagen type I (10 μg/mL) to evaluate melanospheres formation. Data are shown as the mean ± SD (*n* = 3). *P < 0.05; **P < 0.01; ***P < 0.001, one-way ANOVA, post hoc comparisons, Tukey’s test for results in (**d**, **f**, **g**, **h** and **k**)

Studies have demonstrated that ECM remodeling in the host organ can create a favorable microenvironment (niche) which is critical for colonization of tumor cells.^17^ HSCs, one of the most important stromal cell types in liver, are an important source of ECM remodeling impetus in hepatic fibrosis and carcinoma.^18^ We thus wondered whether UM cells were capable of activating HSCs to favor colonization in liver. Exposure of HSCs to the conditioned medium (CM) prepared from co-cultured UM cells with HSCs (direct or indirect as illustrated in Fig. 5a) elicited activation of HSCs evidenced by increased α-SMA as examined with immunofluoresence staining and western blotting analysis (Fig. 5b, c). By contrast, the CM derived from mono-cultured HSCs using either HSC-specific medium or RPMI1640 medium did not induce such an activating effect on HSCs (Fig. 5b, c). These results suggest that the UM cells may secret soluble factor(s) to activate HSCs.

We next collected the CM from the activated HSCs to the culture of melanosphere of UM cells transduced with either Scramble or shDDR1. The results showed that the CM from the activated HSCs induced by the CM from UM cells significantly elevated ability of melanosphere formation in UM cells transduced with lentiviral vector (Fig. 5d). In contrast, the CM from the activated HSCs did not elevate ability of melanosphere formation of UM cells transduced with DDR1 shRNA (Fig. 5d). Western blotting analysis of whole-cell lysates of these UM cells showed that HSCs-derived CM pretreated with UM cells-derived CM led to increased phosphorylation in DDR1 and STAT3 in UM cells (Fig. 5e), suggesting that HSCs may be activated to release soluble factors to ligate membrane receptor DDR1 on UM cells in response to soluble factors derived from UM cells.

### Collagen I is an active factor in the CM from the activated HSC

Linking that HSCs can be activated into contractile myofibroblasts to secret collagens into ECM,^19^ we, therefore, decide to determine the pro-collagen I content in the HSCs CM. ELISA assay results showed an increase of pro-collagen I content in the CM from HSCs that only pre-co-cultured with UM cells or pretreated with UM cells-derived CM when compared with that in the CM from mono-cultured HSCs (Fig. 5f). These results support that our hypothesis that UM cells generate certain soluble factor(s) to activate HSCs secretion, which may trigger ECM remodeling.

To further verify the requirement of collagen I, CM from the HSCs depleted collagen I by shRNA was exposed to UM cells. Western blotting analysis indicated that CM derived from collagen I-depleted HSCs caused less phosphorylation in DDR1 and STAT3 as well as reduced the self-renewal capacity of CSCs in UM cells compared with the CM prepared from Scramble HSCs (Fig. 5g–i).

We next examined whether the in vitro artificial collagen I recapitulated the effect of HSCs CM. The results indicated that addition of recombinant human collagen I to the culture obviously led to phosphorylation in DDR1 and STAT3 in UM cells (Fig. 5j). Furthermore, treatment with recombinant human collagen I significantly elevated the capacity of melanosphere formation in UM cells stably transduced with Scramble lentivirus, but not in UM cells stably transduced with shDDR1 lentivirus (Fig. 5k). These results reveal that the promoting effect of collagen I on self-renewal of CSCs was exerted via DDR1.

### TGF-β1 secreted from UM cells activates HSCs to release collagen I

**Fig. 6:**
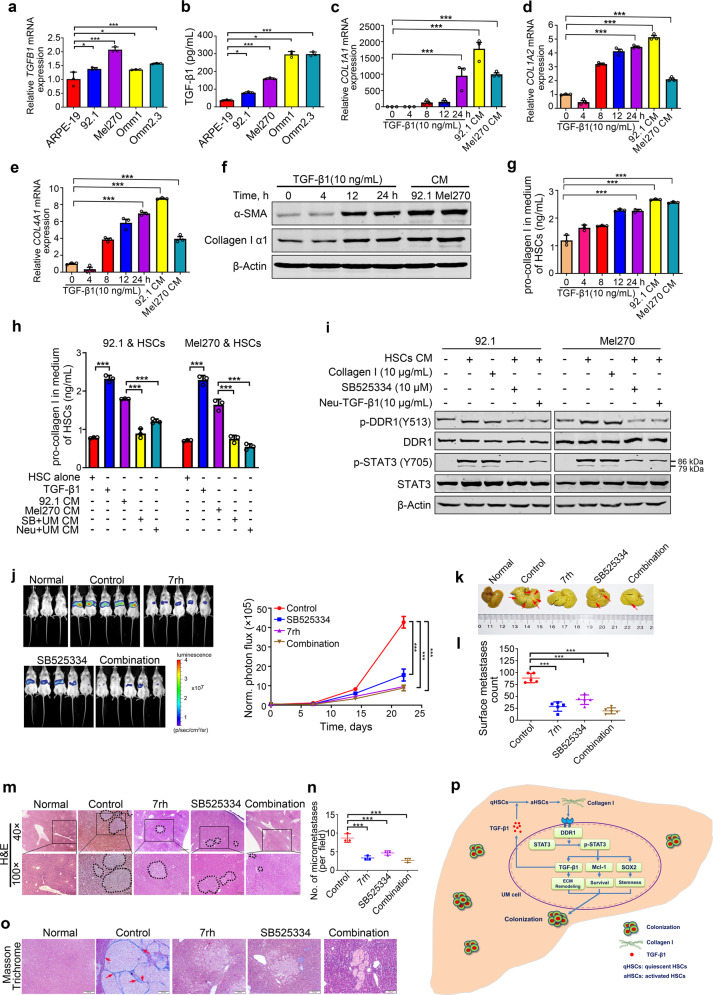
TGF-β1 secreted by UM cells activates microenvironmental HSCs to release collagen type Iα which in turn ligates cell surface DDR1 on UM cells. **a** Expression of TGF-β1 was analyzed by qRT-PCR assay in UM cells and ARPE-19 cells. Data are shown as the mean ± SD (*n* = 3). **b** ELISA analysis of TGF-β1 in the CM of UM cells and ARPE-19 cells is shown. Data are shown as the mean ± SD (*n* = 3). **c**–**e** qRT-PCR analysis of collagen Iα1 (**c**), collagen Iα2 (**d**) and collagen IV (**e**) in HSCs after treated with recombinant TGF-β1 (10 ng/mL) for indicated time periods or CM isolated from 92.1 and Mel270 cells for 6 h, then replaced with fresh HSC medium for 24 h. Data are shown as the mean ± SD (*n* = 3). **f**, **g** HSCs were cultured in the presence of 10 ng/mL recombinant TGF-β1 at different time points or the CM derived from 92.1 and Mel270 cells for 6 h, then replaced with fresh HSC medium for 24 h, The cells were pelleted by centrifugation to extract whole-cell lysates for western blotting analysis (**f**) and the corresponding supernatants were analyzed levels of pro-collagen I using ELISA assay (**g**). Data are shown as the mean ± SD (*n* = 3). **h**, **i** HSCs were treated with recombinant human TGF-β1 (10 ng/mL), or UM-derived CM in the presence or absence of neutralizing anti-TGF-β1 antibody or SB525334 (TGFβRI inhibitor) for 6 h, then replaced with fresh HSC medium for another 24 h. The collagen type Iα in the resultant medium was determined by ELISA assay (**h**). The resultant medium or HSC-derived CM was added into the culture of 92.1 and Mel270 cells for 12 h, and then subjected to western blotting analysis of DDR1 and its signaling molecules (**i**). Data are shown as the mean ± SD (*n* = 3). **j**–**n** After 5 × 10^5^ Mel270-luc cells were intrasplenically inoculated, the NOG mice were administered with vehicle (ddH2O:DMSO:EtOH:Cremophor EL = 90:2:4:4), 7rh (25 mg/kg, orally), SB525334 (30 mg/kg/day, i.p.) alone or combination 7rh (25 mg/kg, orally) with SB525334 (30 mg/kg/day, i.p.) every day for 21 days (*n* = 5 per group). **j** Representative images of luciferase signals on day 21 after treatment with vehicle, 7rh, SB525334 alone or combination 7rh with SB525334 (left). Quantitative analysis of photon flux for hepatic metastases in NOG mice was performed every week *(right)*. Data are shown as the mean ± SD (*n* = 5). The mice were sacrificed to count metastatic nodules on liver surface. Representative images (**k**) and quantitative analysis (**l**) of liver nodules are shown. Data are shown as the mean ± SD (*n* = 5). **m**, **n** Metastasis nodules in paraffin sections of liver tissue from each group were identified by H&E staining. Scale bar: 500 µm (40×), 200 µm (100×). Data are shown as the mean ± SD (*n* = 3). **o** Collagens were detected by Masson trichrome staining in paraffin sections of liver tissue. Scale bar: 200 µm (100×). **p** A proposed working model is shown. UM cells secrete TGF-β1 which induces quiescent hepatic stellate cells (qHSCs) into activated HSCs (aHSCs) which secretes collagen type I in turn activates DDR1, strengthening survival through upregulating STAT3-dependent Mcl-1, strengthening stemness via upregulating STAT3-dependent SOX2, and clonogenicity in cancer cells and ultimately promotes liver colonization and metastasis in UM. *P < 0.05; **P < 0.01; ***P < 0.001, one-way ANOVA, post hoc comparisons, Tukey’s test for results in (**a**–**e**, **g**, **h**, **j**, **l** and **n**)

To define the specific factor(s) in the UM-derived CM activated HSCs, the expression of the cytokines (e.g., PDGFA, PDGFC, IGF, and Endothelin) that are critical for activation of HSCs was detected by qRT-PCR. The results indicated that only TGF-β1 was higher in UM cells than that in ARPE-19 cells (Fig. 6a and Supplementary Fig. S6a–d). Considering that TGF-β1 is the most important cytokine which can activate quiescent HSCs into myofibroblast,^19^ and the transcription of TGF-β1 gene can be regulated by DDR1-STAT3 axis (Supplementary Fig. S7), we paid our attention to TGF-β1. Similarly, the increased content of TGF-β1 was detected in the CM of UM cells compared with that in ARPE-19 cells using ELISA assay (Fig. 6b).

Exposure of HSCs to recombinant human TGF-β1 as well as the CM from UM cells dramatically elicited 1000-fold increase in the expression of collagen Iα1 with limited increase in the expression of collagen Iα2 and collagen IV (Fig. 6c–e). Given that collagen I is the major type of collagen produced by the activated HSCs in response to TGF-β1, and that collagen I is an important ligand of DDR1. The significant elevation of collagen Iα1 was further confirmed in HSCs after exposure to recombinant human TGF-β1 or the CM from UM cells with western blotting assay (Fig. 6f). ELISA assay analysis of the CM from HSCs stimulated by recombinant human TGF-β1 as well as the CM from UM cells further validated a significant upregulation in pro-collagen I, which was abrogated in the presence of TGF-β1 inhibitor, SB525334 or anti-TGF-β1 neutralizing antibody (Fig. 6g, h). Consistently, western blotting results showed that the DDR1 activation was attenuated in UM cells treated with HSC CM from TGF-β1 inhibitor, SB525334 or anti-TGF-β1 neutralizing antibody group when compared with the non-treated HSC CM or recombinant collagen I stimulation (Fig. 6i). These results support a tumor cells-microenvironment interaction loop that TGF-β1 secreted from UM cells activates stromal HSCs to release collagen I which in turn ligate cell surface DDR1 on UM cells.

### Interrupting the TGF-β1-collagen I-DDR1-STAT3 loop abrogates liver metastatic colonization in UM

The tumor cells-microenvironment interaction loop predicted that blockade of either TGF-β1 signaling in microenvironmental HSCs or DDR1 signaling in UM cells might inhibit in vivo metastatic colonization. To test this hypothesis, the NOG mice received intrasplenic injection of Mel270-luc or Omm2.3-luc cells were administrated with 7rh, SB525334 or combination. The mice treated with vehicle manifested strong bioluminescence signal in approximately liver area (Fig. 6j and Supplementary Fig. S8a, b) and increased numbers and size of metastatic nodules on liver (Fig. 6k, l and Supplementary Fig. S8c). In a sharp contrast, the mice administrated with either 7rh or SB525334 manifested much less bioluminescence signal intensity in the liver area (Fig. 6j and Supplementary Fig. S8a, b) and numbers of metastatic nodules on liver surface (Fig. 6k, l and Supplementary Fig. S8c). In accordance, H&E staining showed an obvious reduction in density and size of the metastatic nodules in the livers of treated group relative to those in non-treated group (Fig. 6m, n and Supplementary Fig. S8d, e). Of note, the efficacy that 7rh or SB525334 inhibited the liver metastasis of UM was similar to the combinational treatment with 7rh and SB525334, suggesting that they did not produce synergy or additive anti-metastasis effect. The results of Masson Trichrome staining revealed large thick bundles of collagen surrounding tumor cell nests to form a niche in liver with metastasis compared with the normal liver, and collagen was effectively decreased in the liver by 7rh, SB525334 or combination treatment (Fig. 6o and Supplementary Fig. S8f). These findings indicate that UM cells in liver may interact with HSCs, and activate HSCs to secret collagen I, forming a beneficial niche for colonization of UM cells (Fig. 6p).

## Discussion

Little is known about the mechanism of metastatic colonization. In the present study, we found that DDR1 is overexpressed in the cancerous cells and primary tissues of UM and promotes cellular proliferation and survival. Pharmacological inactivation of DDR1 tyrosine kinase impedes outgrowth of xenografted Omm1 cells and PDX in NOD-SCID mice. Mechanistically, DDR1 confers maintenance of CSCs traits via STAT3-dependent SOX2 upregulation in UM cells. Further, co-culture experiments show that UM cells secrete TGF-β1 which in turn induces HSCs to produce collagen type Iα1, remodeling ECM. DDR1 responds to collagen I, resulting in strengthened survival, proliferation, and stemness in cancer cells, and promoting metastatic colonization in liver.

### DDR1 is highly expressed in UM

DDRs, unique tyrosine kinase receptors that function as a sensor ECM, are implicated in a wide range of cellular processes.^20^ Our results show that DDR1 rather than DDR2 is overexpressed in UM cells as well as specimens from patients with UM, which is in line with the previous studies that DDR1 expression is restricted to epithelial cells in solid tissues.^21^ DDR1 is reported to be also overexpressed in melanoma arising from cutaneous melanocytes.^22^ In addition, DDR1 is overexpressed in prostate cancer, glioma, and human hepatocellular carcinoma.^20^

In the present study, we demonstrated that targeting DDR1 by shRNA or inhibitor 7rh, restrained proliferation, survival and stemness of UM cells. Pharmacological inactivation of DDR1 tyrosine kinase impedes outgrowth of xenografted Omm1 cells and PDX in NOD-SCID mice. Given that DDR1 is actually not essential because DDR1-null mice does not impair embryonic development,^23^ it is likely that tumor cells may become addicted to the increased levels of oncogenic DDR1. The ECM remodeling such as collagens accumulation in the microenvironment can easily lead to activation of DDR1. Of note, unlike the pattern of instantaneous activation and fast fading in soluble cytokine binding receptor tyrosine kinases, collagen binding to DDR1 eliciting a slow and sustainable activation pattern lasting even up to 18 h.^24^ At the present time, it’s not clear about the underlying mechanism that DDR1 is overexpressed in UM. TCGA analysis showed that amplification is the dominant alternation in DDR1, accounting for only ~10% cases of UM.^25^ Other potential mechanisms (e.g., miRNA regulation, protein stability alteration) for deregulated DDR1 need to be further clarified.

### DDR1 promotes cellular survival of cancer cells

Enhanced survival capability, an important hallmark of cancer, is critical for outgrowth of cancer metastasis. One of the principal mechanisms of pro-survivial or resistance to apoptosis in cancer is the deregulation of members of the Bcl-2 protein family which controls the integrity of the outer mitochondrial membrane.^26^ Mcl-1, a member of the Bcl-2 protein family, has been shown to be up-regulated in numerous hematological and solid tumor malignancies.^27^ Its overexpression positively correlates with tumor grade and resistance to standard chemotherapy.^27^ Loss of cell viability was observed in response to siRNA-induced reduction of Mcl-1 or small-molecule inhibitor targeting transcription of Mcl-1 in cancer cells.^28^ Additionally, some kinase inhibitors are believed to exert their antiproliferative effects by indirectly downregulation of Mcl-1 mRNA via inhibition of RNA polymerase II-dependent transcription inhibition.^28^

Consistent with previous studies, our results showed that targeting DDR1 with 7rh induced apoptosis with decline in Mcl-1 transcription. Mcl-1 has been reported a target gene of STAT3.^13^ Plausibly, the presence of STAT3 binding sites in the promoter of Mcl-1 was confirmed by using ChIP assay in this study; the results reveal that the repressed Mcl-1 transcription during the 7rh-induced apoptosis may be via DDR1-STAT3 axis. Therefore, our results support that inhibition of Mcl-1 may offer a therapeutic target in metastatic UM.

### DDR1 facilitates the traits of CSCs in UM

In our study, we found that targeting DDR1 by 7rh or shRNA led to decrease in ALDH^+^ cells, ability of melanosphere formation and serially-replating, and in vivo CSCs frequency. Further study showed that among the stemness-related proteins, SOX2 was reduced in the DDR1-depleted UM cells. With ChIP assay, STAT3 was involved in the DDR1 knockdown-enabled SOX2 transcription downregulation. Therefore, it’s reasonable to postulate DDR1 facilitates the traits of CSCs involved in STAT3 mediated-SOX2 transcription in UM. SOX2, a protein initially is known as a regulator of self-renewal in mouse and human embryonic stem cells, as well function as a key transcription factor to induce pluripotent stem cells from fibroblast cells.^29^ Mounting evidence suggests that SOX2 is a critical biomarker of self-renewal and maintains the stemness of CSCs in types of cancer, including breast cancer, osteosarcomas and glioma.^30^

### ECM remodeling and metastasis

Almost every cell in the body is exposed to ECM, a complex and dynamic network of macromolecules with different physical and biochemical properties.^31^ ECM is not just an inert supportive scaffold, but regulates diverse cellular behaviors.^31^ Of importance, ECM is a dynamic compartment because of alternation in production, degradation, and remodeling of its components.^31^ Alteration in a specific ECM component may impact on the biochemical, biomechanical, and physical properties of the ECM, leading to disorganized network and eventually remodeling.^32^ Collagens accounting for 30% of body proteins are a major type of component of ECM^31^. In liver disease, HSCs are particularly vigorous compared with the rest types of residue cells (e.g., hepatocytes and liver sinusoidal endothelial cells). In the pathogenesis of virus-associated hepatic fibrosis or cirrhosis, a critical step is the activation of HSCs from the quiescent state leading to collagen excessive accumulation.^33^ In this study, progressive and excessive accumulation in collagens was observed in the tumor cell-infiltrated liver of NOG mice (Supplementary Fig. S9), which is consistent with the previous finding that increased number of activated HSCs and accumulated collagen existed in the hepatectomy samples of patients with UM.^10^ Experiments of liver metastasis also showed that co-inoculation of HSCs increased the number of UM hepatic metastases.^10^ Our further analysis suggested that collagen Iα1 is the major type of collagens that HSCs secreted when they are exposed to CM of UM cells. In accord with this, HSCs secretes collagen I in the presence of hepatoma cells.^34^ Because collagen Iα1 is a potent ligand of DDR1 receptor, it’s plausible to postulate that ECM remodeling featured with excessive collagen Iα1 elicits sustaining activation of DDR1 and metastatic colonization.

The possibility of involvement of integrin receptors in the TGF-β1-collagen I loop cannot be excluded at the present time. It has been reported that integrins such as collagen receptor α1β1 are expressed in UM cells.^35^ ECM collagen I can bind cell surface integrin α1β1, likely either activating STAT3 in a Src-dependent manner or cross-talking with DDR1 signalling pathways,^36^ and thereby being involved the TGF-β1-collagen I loop.

### Tumor cells-derived TGF-β1 activates HSCs

In the hepatectomy tissues from patients with UM, activated HSCs and their pathological matrix were observed surrounding the malignant foci.^37^ It’s well known that TGF-β1 is a major potent profibrogenic cytokine to activate HSCs. TGF-β1 employs SMAD pathway that induces expression of the genes needed for resting HSCs activation to activated HSCs,^38^ TGF-β1 plays a critical role in promoting collagen secretion and upregulation of MMPs in activated-HSCs, which can exacerbate the malignant phenotype and lead to increased invasion and subsequent metastasis.^39^ Targeting TGF-β1 may be a promising strategy for management of metastasis by restricting ECM activation and attenuating collagen secretion.

Overexpression of TGF-β is commonly observed in solid tumors.^40^ Because transformed cells (e.g., esophageal cancer, gastric cancer, hepatocellular carcinoma) can secret cytokines,^41^ we measured the expression of cytokines (e.g., TGF-β1, PDGFA, PDGFC, IGF, and Endothelin) of UM cells and found that the expression of TGF-β1 was increased. ELISA assay also showed that TGF-β1 was increased in the conditioned medium of UM cells. However, the mechanism of the high expression of TGF-β in tumor cells remains unclear. Some studies showed that abnormal DNA methylation on its promoter may lead to its anomalous transcription of TGF-β.^42^ The mechanism of overexpression of TGF-β in tumor cells requires further study.

### DDR1 is a druggable target independent of BAP1 in UM

Recently, mutations of several genes such as GNAQ, GNA11, BAP1, SF3B1, EIF1AX, PLCB4, and CYSLTR2 have been identified in UM.^43^ In particular, loss-of-function mutations of BAP1 gene are found to be associated with a poor outcome in 84% of metastatic UM patients, hinting a tumor suppressor role of BAP1 in metastasis. However, the context of BAP1 signaling pathway in UM metastasis remains unclear.^44^ UM cells used for metastatic experiments in this study harbor wild-type BAP1 (Supplementary Fig. S10a, b), our results showed that DDR1 deletion did not affect the expression of BAP1 (Supplementary Fig. S10c). On the other hand, knockdown of BAP1 did not change the protein levels of DDR1 and its downstream signal pathway (Supplementary Fig. S10d). These results reveal that the expression of DDR1 and BAP1 may be independent in UM cells.

### TGF-β inhibitors and DDR inhibitors

In our study, the results that using SB525334 to block TGF-β1 signaling interrupted the TGF-β1-collagen I-DDR1 loop and inhibited liver metastasis in UM. Because the anti-TGF-β1-based therapy is an attractive therapeutic target of microenvironment, novel drugs blocking the TGF-β pathway, including blocking production of TGF-β ligands with antisense molecules, small-molecule inhibitors of the kinase activity of TGFβRI and TGFβRII, monoclonal antibodies that block TGF-β signaling, and soluble forms of TGFβRII and TGFβRIII that function as ligand traps, have been developed and have shown efficacy in preclinical and clinical studies.^45^ More than 100 clinical trials (https://clinicaltrials.gov) in which the effect of TGF-β has been studied in patients with different diseases are undergoing. The first clinical human dose-escalation study in solid cancer (e.g., melanoma, breast cancer, hepatocellular carcinoma, and prostate cancer) patients (NCT02160106) targeting TGFβRI with TEW-7197 causing downregulation of TGF-β signaling is currently completed with encouraging results. This line of progress may help clinical trials of TGF-β inhibitors in prevention and treatment in patients with metastatic UM in future.

The concentration of 7rh required for a pharmacodynamic or antiproliferative effect sounds higher in UM cells in the present study than other types of carcinoma (e.g., nasopharyngeal carcinoma, pancreatic cancer).^46,47^ The differential sensitivity may due to discrepancy in cell type context and culture system.

Apart from 7rh, other promising small-molecule inhibitors of DDR1 have been developed.^12^ Treatment with 7rh or SB525334 exhibited negligible toxicity to the mice as reflected by body weight changes (Supplementary Fig. S11). Targeting DDR1 in combination with targeting microenvironment TGF-β may a new attractive approach for treatment of UM liver metastasis.

In conclusion, our results reveal that ECM remodeling activates the overexpressed extracellular matrix collagen receptor DDR1 promoting colonization phenotypes (e.g., survival, outgrowth and stemness of cancer cells in the metastatic site). The UM cells secret cytokine TGF-β1 activating HSCs to secret collagen I, remodeling ECM and forming a positive feedback loop. We, therefore, propose a working model (Fig. 6p). Targeting DDR1 signaling and TGF-β1 signaling may be a novel approach to diminish hepatic metastasis in UM.

## Materials and methods

### Chemicals and antibodies

DDR1 inhibitor 7rh (chemical structure, Fig. 1i) was synthesized in our laboratory.^12^ TGFβRI inhibitor SB525334 was obtained from Selleck (Shanghai, China). Annexin V-FITC and collagen I were from Sigma-Aldrich (Shanghai, China). Recombinant TGF-β1 was purchased from Invitrogen (Shanghai, China). Antibodies against DDR1, Survivin, and Bcl-X_L_ were from Santa Cruz Biotech (Santa Cruz, CA). Antibodies against PARP (clone 4C10-5), caspase-3, XIAP, and Bcl-2 were purchased from BD Biosciences (San Jose, CA). Antibodies against DDR2, STAT3, p-STAT3 (Y705), p-DDR1 (Y513), active Caspase-3, Bax, α-SMA, SOX2, NANOG, OCT4, KLF4, SLUG and ALDH were from Cell Signaling Technology (Beverly, MA). Anti-β-actin was from Sigma-Aldrich (Shanghai, China). Secondary antibodies used were fluorescent-conjugated anti-mouse IgG and anti-rabbit IgG (LI-COR Biotechnology, Nebraska, USA).

### Cell culture

The UM cell lines (92.1, Mel270, Omm1, and Omm2.3) were generously provided by Dr. M.J Jager (Leiden University Medical Center, Leiden, the Netherlands) and cultured in RPMI1640 supplemented with 10% FBS (Biological Industries, Kibbutz Beit Haemek, Israel). Human adult retinal pigmented epithelium (ARPE-19) cells and MP41 cells were purchased from American Type Culture Collection (ATCC, Manassas, VA) cultured in RPMI1640 supplemented with 10% FBS and 20% FBS, respectively.^48^ Hepatic stellate cells LX-2 obtained from ATCC were cultured in stellate cell medium (Sciencell, San Diego, CA) supplemented with 2% FBS and specific cytokines. For packaging virus, human embryonic kidney 293 T cells were obtained from ATCC grown in DMEM medium with 10% FBS. All cells were kept in a 37 °C humidified incubator in the presence of 5% CO_2_.

### Cell viability assay

MTS assay was used to measure cell viability as previously described.^49^ Briefly, UM cells were seeded at a density of 5000 cells per well into 96-well plates and treated with escalating concentrations of 7rh from 0 to 20 μM. After 68 h, 20 μl MTS (CellTiter 96 Aqueous One Solution reagent; Promega) was added into each well and incubated for another 4 h. Absorbance was recorded at 490 nm with a Synergy HT Microplate Reader (Bio Tek) and IC_50_ value was calculated via curve fitting of the sigmoidal dose–response curve (GraphPad Prism 5, La Jolla, CA).

### Colony-formation assays

Double layer soft agar system was utilized to evaluate the colony-forming capacity of UM cells as previously described.^49^ Briefly, UM cells were pretreated with 7rh (0, 5, 7.5, 10 μM) for 24 h and plated at 5000 cells/well resuspended in complete RPMI1640 medium containing 0.5% agar, and seeded over a layer of 1% agar in 24-well plates for 2 weeks at 37 °C. Colonies composed of >50 cells were counted using an inverted phase-contrast microscope.

### Real-time quantitative RT-PCR

Total mRNA from cells or tissues was prepared using Trizol reagent (Invitrogen). The first-strand complementary DNA (cDNA) was synthesized with maxima first-strand cDNA synthesis kit (Thermo Fisher). According to the manufacturer’s instructions, the qRT-PCR reaction was carried on a BIO-RAD CFX96 Real-Time Thermocycler (CFX96, Bio-Rad Laboratories, Hercules, CA) with SYBR Premix Ex Taq (Perfect Real-time; Takara Bio). Relative expression of each gene was normalized to GAPDH by utilizing the 2^−∆∆CT^ method, where ∆∆C_T_ = (C_T, Target gene_ − C_T, GAPDH_) _treated cells_ − (C_T, Target gene_ − C_T, GAPDH_) _control cells_. PCR primers were listed in Supplementary Table S4.

### Western blotting analysis

Whole-cell lysates were prepared by sonication in cold RIPA buffer containing 1× protease inhibitor cocktail (Roche), 10 mM β-glycerophosphate, 1 mM sodium orthovanadate, 10 mM sodium fluoride, and 1 mM phenylmetnylsulfonyl fluoride. Equal amounts of protein samples were separated by SDS-PAGE gel electrophoresis and transferred to nitrocellulose membranes, then incubated with the primary antibodies overnight. The β-actin was used as the internal control of protein loading, and the immunoblots were recorded with the Odyssey infrared imaging system (LI-COR) after exposure to appropriate IRDye 680CW or IRDye 800CW secondary antibodies (LI-COR, Lincoln, Nebraska) for 1 h at room temperature.^49^

### Apoptosis assay by flow cytometry

Apoptosis were conducted using Annexin V-FITC (fluoroisothiocyanate) kit (Sigma-Aldrich, Shanghai) as previously described.^49^ Briefly, after UM cells exposed to elevating concentration of 7rh (0, 5, 10, 15 μM) for 48 h or 15 μM 7rh for different durations (0, 24, 36, 48 h), 2 × 10^5^ cells were collected and resuspended in 100 μl binding buffer (10 mM HEPES/NaOH [pH7.4], 1 M CaCl_2_, 4 M NaCl) mixed with 0.3 μL Annexin V-FITC. After incubation for 15 min at room temperature in dark, the cell pellets were washed and another 0.5 mL of binding buffer was added. Apoptosis was then analyzed by flow cytometer immediately after staining with 1 μL propidium iodide (Sigma-Aldrich, Shanghai).^49^

### Measurement of mitochondrial transmembrane potential

The mitochondrial transmembrane potential (ΔΨm) in UM cells were measured by flow cytometry with molecular probes (Invitrogen). In brief, cells were preincubated with 15 μM 7rh for the indicated time (0, 24, 36, 48 h). 1 × 10^5^ cells were then harvested to load with 0.3 μM CMXRos and 0.1 μM MTGreen (MitoTracker probes) at 37 °C for 1 h in dark and washed with PBS. Changes in ΔΨm were subjected to analysis with flow cytometry.^49^

### Aldehyde dehydrogenase (ALDH) assay

The ALDH enzymatic activity of 7rh-treated UM cells was measured via using Aldefluor kit (Stem Cell Technologies, Vancouver, BC, Canada) according to the instructions of the manufacturer as previously described.^50^ In short, after treated with 10 μM 7rh for 48 h, 1 × 10^5^ UM cells were harvested and resuspended in 500 μL ALDH assay buffer containing 5 μL ALDH reagent with or without 5 μL DEAB at 37 °C for 1 h in dark. The cells were then washed with PBS and subjected to ALDH activity analysis by flow cytometery.^49^

### Melanosphere culture

After treated with 10 μM 7rh for 24 h, UM cells were collected and seeded at a density of 5000 cells each well in 24-well Corning^TM^ Ultra-Low Attachment Plates (Thermo Fisher Scientific Inc., Waltham, MA). Medium used was DMEM/F-12 medium (HyClone) containing 1 × B27, 10 ng/mL bFGF, 20 ng/mL EGF. Cultures were fed every two days and passaged weekly. On day 7 of culture, the melanospheres were harvested, dispersed, and re-cultured with the same culture medium in low-attachment 24-well plates for the secondary and tertiary rounds of tumor sphere culture. The number of melanosphere (cells >50) in each well was manually counted with an inverted microscope for each round.^49^

### Chromatin immunoprecipitation (ChIP) assay

According to the instructions of the manufacturer, ChIP assay was performed using the EZ-ChIP Kit (EMD Millipore) as previous procedure.^49^ Briefly, 1 × 10^7^ Mel270 cells were collected and cross-linked by formaldehyde for 10 min followed by quenched with glycine. The cells were washed, lysed, and subjected to sonication to shear the cross-linked DNA fragments to 200–1000 bp in length. Anti-STAT3 antibody (1:200) or normal rabbit IgG (negative control) were immunoprecipitated with the cross-linked protein-DNA overnight at 4 °C with rotation. Immunocomplexes were collected, washed, and DNA was eluted. The cross-links of protein-DNA complexes were reversed overnight, and protein and RNA were digested with proteinase K and RNase, respectively. DNA was purified with spin columns and subjected to qRT-PCR assay. The primers for amplification of purified DNA were listed in Supplementary Table S5.

### HSCs CM collection

When 92.1 or Mel270 cells reached ~80% confluence in flask, cells were washed with PBS, and serum-free RPMI1640 medium was replenished. Twelve or 24 h later, the supernatant (designated CM of UM) was collected followed by centrifugation at 14,000 rpm. The CM of UM was added to the starved HSCs for 6 h and replaced with fresh stellate cell medium and collected the medium at 12 h and 24 h as HSCs CM.

### Immunofluorescence staining

After treatment with different media grown on six-well coverslips plates, HSCs were fixed with 4% paraformaldehyde for 20 min and permeabilized with 1% Triton X-100 plus 0.5% NP-40 for 15 min. The cells were blocked with 5% bovine serum albumin (BSA) for 1 h at room temperature followed by staining with anti-α-SMA antibody (1:100) overnight at 4 °C. Cells were then washed, incubated with the corresponding fluorescent secondary antibody (1:1000) for 1 h at room temperature, and loaded with DAPI for 5 min. Finally, anti-fading agent was added to cover slips. The confocal microscope was employed to observe immunofluorescence images.^50^

### Enzyme-linked immunosorbent assay (ELISA)

According to the manufacturer’s instructions, the protein content of Pro-Collagen Iα in HSC CM or TGF-β1 in UM CM was detected by using human Pro-Collagen Iα or TGF-β1 Quantikine ELISA Kit (R&D Systems, Minneapolis), respectively. Briefly, HSC CM or UM CM was added to the defined wells of ELISA plates that were pre-coated with specific monoclonal antibody for 2 h at room temperature. Subsequently, plates were washed three times with PBS to remove the unbound substances, and then enzyme-linked human Pro-Collagen Iα or TGF-β1 polyclonal antibody was added for incubation another 2 h at room temperature. Double distilled H_2_O was used to wash away the unbound antibody-enzyme reagent, and finally, substrate solution was added for incubation another 30 min. The optical density was measured at 450 nm after the color development was stopped.^50^

### Retrovirus and lentivirus infection

pCMV empty vector, constructs encoding full-length human Mcl-1, pTSB-CMV and pTSB-CMV-STAT3 were from Transheep (Shanghai, China). Non-targeting (mock) siRNA and siRNA oligoduplexes against Mcl-1 were from Dharmacon RNA Tech (Lafayette, CO). Scramble (pLKO.1-puro-Non-target shRNA), human DDR1 specific target shRNA (pLKO.1-puro-DDR1-target shRNA), human SOX2 specific target shRNA (pLKO.1-puro-SOX2-target shRNA), human Collagen I specific target shRNA (pLKO.1-puro-Collagen I-target shRNA) were from Sigma-Aldrich (Shanghai, China). pBABE-puro-DDR1 and pBABE-puro-DDR1(P529*) were provided by Dr. Hua Gao (Tongji University, Shanghai).^14^ Briefly, for transfection of siRNA duplexes, shRNA constructs and overexpressing plasmids, nucleotides were mixed with polyethylenimine (Polysciences, Warrington, PA) in opti-MEM medium and then added to target cells. For stable knockdown of DDR1, SOX2, Collagen I or overexpression of STAT3, DDR1, and DDR1 (P529*), the lentivirus and retrovirus supernatants were prepared by 293 T cells using the lentivirus packing system [pCMV-dR8.2 (the packing construct), the pCMV-VSVG (envelope construct)] and the PCL retrovirus packing system, respectively. Viral supernatants were collected and purified with 0.45-μm filters at 48 and 72 h after transfection. Then viruses were added to UM cells, and cells were selected with 1 mg/mL puromycin for 1 weeks.^50^

### Primary tissues of UM patients

Primary tissues of UM were obtained from patients underwent primary enucleation or local resection between 2012 and 2016 in Zhongshan Ophthalmic Center of Sun Yat-sen University, after informed consent according to the principles of Helsinki Declaration. The tumor tissues were stained with anti-DDR1 antibody (1:100) and scored. The ratio of positive-staining cells was scored as follows: 0 (no positive tumor cells), 1 (<10% positive tumor cells), 2 (10–40% positive tumor cells), 3 (<41–70% positive tumor cells), and 4 (>70% positive tumor cells). The staining intensity was measured following the standard: 0 (no staining), 1 (low intensity), 2 (medium intensity), and 3 (high intensity). The staining index (SI) was scored using the proportion of positively stained cells×intensity and defined as negative scored 0, low scored 1–3, medium scored 4–8, and high scored 9–12.^50^

### NOD-SCID mouse xenograft tumor experiment

Omm1 cells (3 × 10^6^ in 200 μl PBS) were inoculated subcutaneously into 4–6-weeks-old male NOD-SCID mice (Vital River Laboratories, Beijing, China). The long and short diameters of the tumor were measured every other day. The tumor volume was calculated according to the formula: V = a^2^ × b × 0.4, where a indicates the short diameter and b indicates the long diameter of the tumor. When the tumor volume reached approximately 100 mm^3^, the mice were randomly divided into 2 groups of 8 mice each. One group treated with vehicle (DMSO: EtOH: Cremophor EL: H2O = 2:4:4:90) and the other group was administered with 7rh (25 mg/kg/day, oral) for 2 weeks. The mice were sacrificed and subcutaneous tumors were removed, weighed, and photographed. DDR1 and its downstream signal in tumor lysates were detected by western blotting. H&E staining and IHC assay were performed on formalin-fixed tumor tissue.^50^

### Patient-derived xenograft model

The effects of 7rh on proliferation of UM were evaluated in patient-derived xenograft (PDX) which was established according to the previous studies.^48,49^ Briefly, 1 × 10^7^ MP41 cells in 200 μL PBS were subcutaneously implanted into the flanks of 4–6-weeks-old male NOD-SCID mice purchased from Vital River Laboratories (Beijing, China). Once tumor volume reached about 800 mm^3^, the tumors were harvested, cut into 30–60 mm^3^ slices, and inoculated into the NOD-SCID mice as aforementioned. After three serial passages in vivo, the tumor xenografts were used for following experiments. Tumor pieces with a volume of 30–60 mm^3^ were subcutaneously grafted into the flanks of the NOD-SCID mice. The mice were randomly divided into vehicle or 7rh groups (*n* = 8 per group) once tumors grown to ~200 mm^3^, and then treated for 22 days as described above. Tumors were isolated from euthanized mice when the volume reached to ~1200 mm^3^.

### Limiting dilution in NOD-SCID mice

Omm1 cells stably expressing Scramble or shDDR1 were counted, and different cell doses per group were subcutaneously transplanted into 4–6-weeks-old male NOD-SCID mice (*n* = 6 per group). On day 35, mice were euthanized and the subcutaneous tumors were dissected and photographed. The CSC frequency in UM cells was examined using L-Calc dilution software (STEM CELL Technologies Inc.).^50^

### Liver metastasis model in NOG mice

Mel270-luciferase cells (5 × 10^5^) or Omm2.3-luciferase cells (1 × 10^6^) were injected into the spleen of 4–6-week-old female NOG mice (Vital River Laboratories, Beijing, China) in 50 μL of sterile PBS (*n* = 5 per group). After the inoculation, mice were treated with 7rh (25 mg/kg/day, oral administration) and SB5253345 (15 mg/kg/day, oral administration) alone or combination. To evaluate tumor metastasis, live imaging of mice was performed weekly with IVIS Lumina II (PerkinElmer). Approximately 28 days later, the mice were euthanized and liver was immediately removed, nodules on the surface were counted. The liver were fixed and subjected to H&E staining. All animal experiments were performed in accordance with the protocol approved by the Sun Yat-sen University Institutional Animal Care and Use Committee guidelines.^50^

### Statistics

All the experiments were performed three times and data were expressed as mean ± standard deviation (SD), unless otherwise stated. Statistical analysis was performed using GraphPad Prism 8.0 software (GraphPad Software, San Diego, CA). Difference between two groups was tested by two-sided Student’s *t* test; the analyses of experiments with multiple groups were performed by one-way analysis of variance (ANOVA) with *post-hoc* intergroup comparisons with the Tukey’s test. *P* < 0.05 was considered to be a statistically significant difference.

## Supplementary information

Supplementary materials and data

## Data Availability

The clinical information of UM specimens can be found in Supplementary Tables 1–2. Detailed information on the sequences of primers can be found in Supplementary Tables 4–5. Other original data in our study are available upon request.
