# Supplementary material for: Activation of transmembrane receptor tyrosine kinase DDR1-STAT3 cascade by extracellular matrix remodeling promotes liver metastatic colonization in uveal melanoma
Source: Signal Transduct Target Ther. 2021 May 12;6:176. doi: 10.1038/s41392-021-00563-x (PMC8113510; doi:10.1038/s41392-021-00563-x)
Supplement: Supplementary file 1 — Supplementary materials and data [file 41392_2021_563_MOESM1_ESM.docx]

Supplementary Materials for

**Activation of transmembrane receptor tyrosine kinase DDR1-STAT3 cascade by extracellular matrix remodeling promotes liver metastatic colonization in uveal melanoma**

Wei Dai^1, 2^, Shenglan Liu^3^, Shubo Wang^1^, Li Zhao^1^, Xiao Yang^1^, Jingfeng Zhou^3^, Yun Wang^1^, Jing Zhang^3^, Ping Zhang^3^, Ke Ding^4^, Yangqiu Li^2^, Jingxuan Pan^3^

^1^Jinan University Institute of Tumor Pharmacology, College of Pharmacy, Jinan University, Guangzhou, China

^2^Integrated Chinese and Western Medicine Postdoctoral Research Station, Jinan University, Guangzhou, China

^3^State Key Laboratory of Ophthalmology, Zhongshan Ophthalmic Center, Sun Yat-sen University, Guangzhou, China

^4^International Cooperative Laboratory of Traditional Chinese Medicine Modernization and Innovative Drug Development of Chinese Ministry of Education (MOE), Guangzhou City Key Laboratory of Precision Chemical Drug Development, College of Pharmacy, Jinan University, Guangzhou, China.

***Correspondence to**: Jingxuan Pan, **email:** [panjx2@mail.sysu.edu.cn](mailto:panjx2@mail.sysu.edu.cn)

**This PDF file includes:**

Supplementary Figure S1 to S11

Supplementary Table S1 to S5

**Supplementary Figure S1**

**
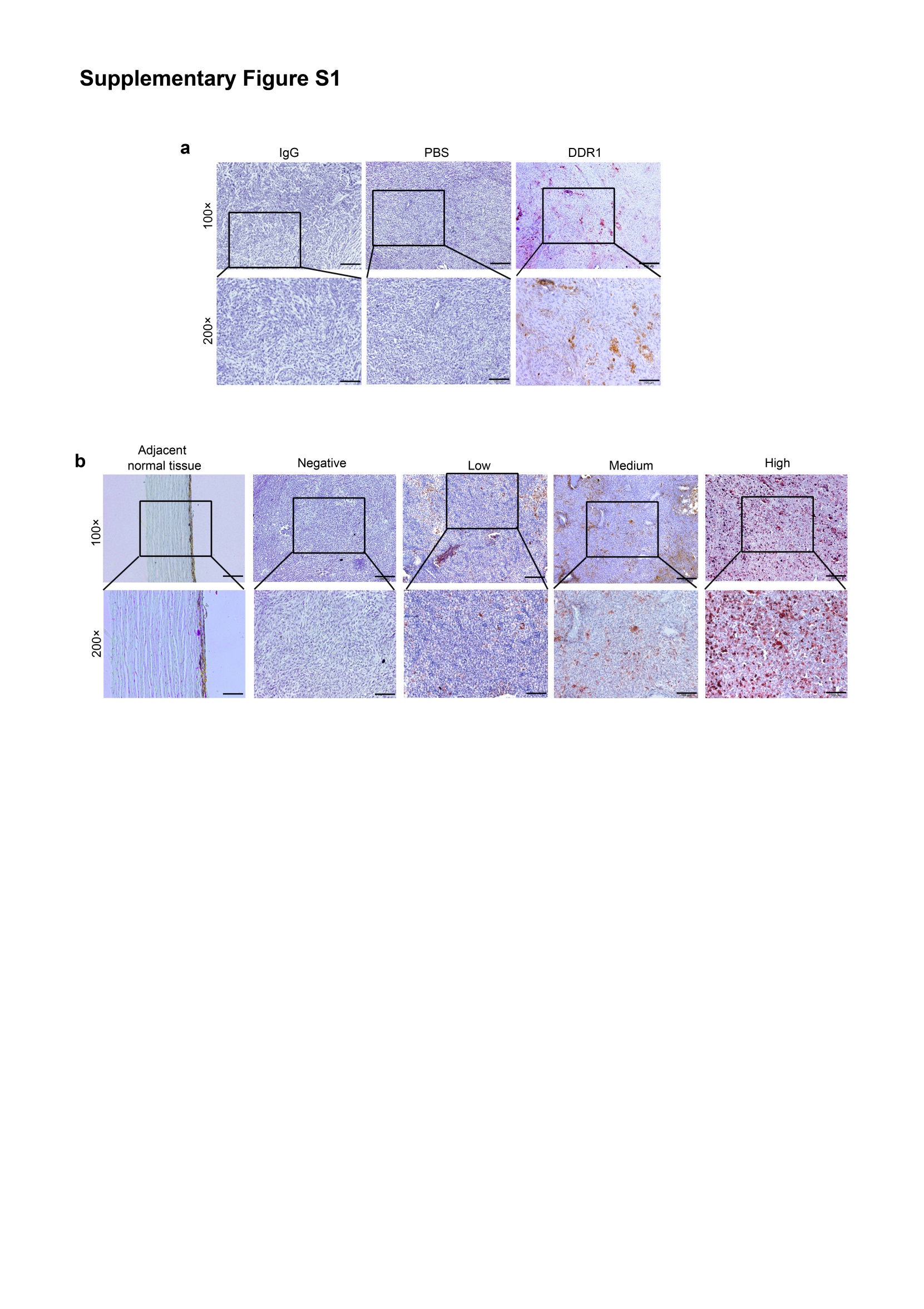
**

**Supplementary Figure S1. DDR1 is highly expressed in paraffin embedded tissues derived from patients with UM relative to adjacent normal tissues. a** The specificity of the anti-DDR1 antibody was examined by IHC staining, PBS and IgG served as negative control. Scale bar: 200 µm (100×), 100 µm (200×). **b** Representative IHC images of DDR1 expression in paraffin-embedded tissues from the patients with UM (n=62) are shown. The staining intensity was scored on four levels (negative, low, medium and high). Scale bar: 200 µm (100×), 100 µm (200×).

**Supplementary Figure S2**

**
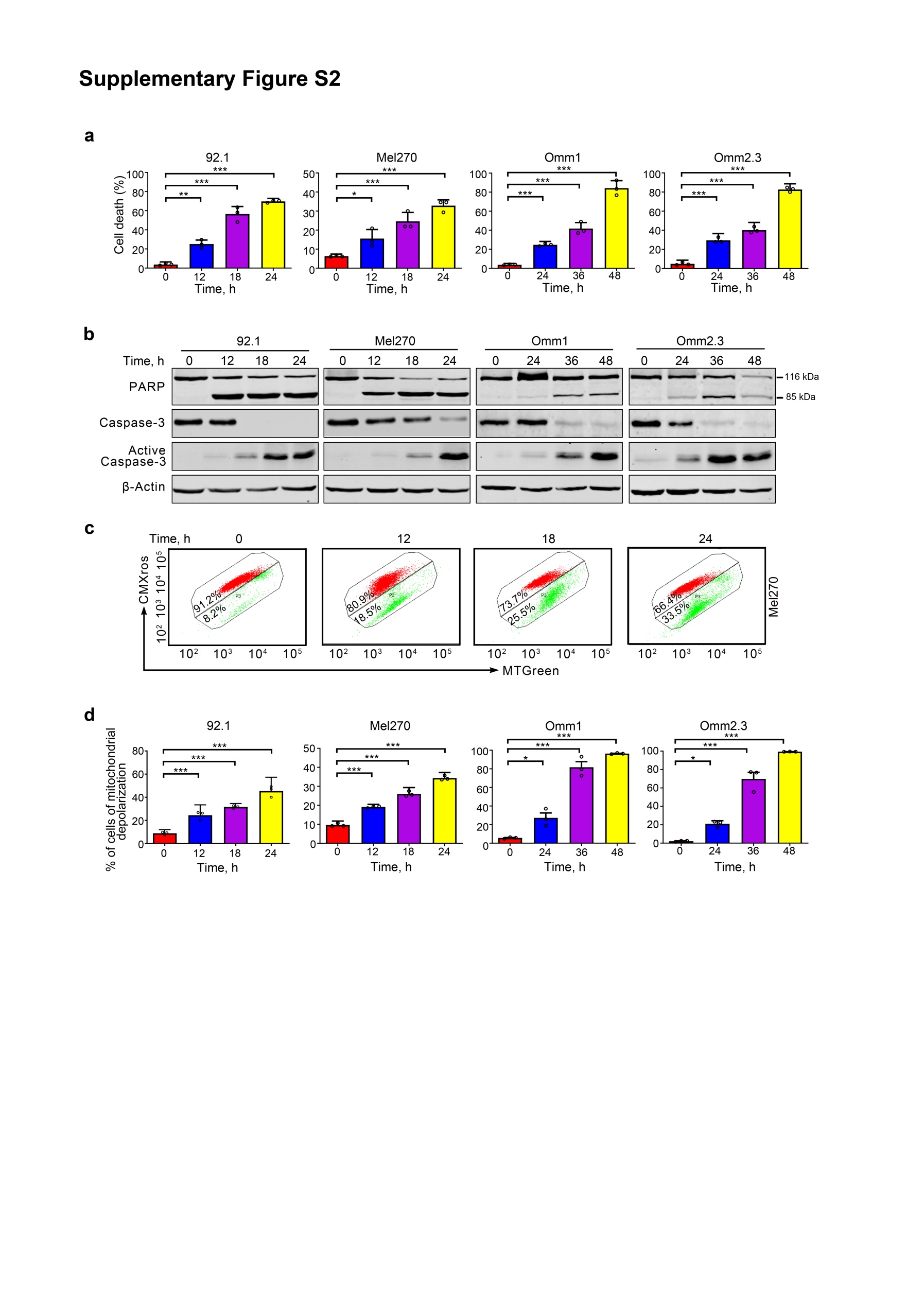
**

**Supplementary Figure S2. 7rh elicits mitochondrial damage and apoptosis in UM cells. a** UM cells were exposed to 15 μM 7rh for the different time periods, flow cytometry was employed to detect the apoptosis after dual-staining with Annexin V-FITC and PI. Results from 3 independent experiments are shown. Data are shown as the mean ± SD (n = 3). **b** Cleavage of PARP and activation of caspase-3 were determined by Western blotting after treatment with 7rh for the different time periods in UM cells. **c, d** UM cells treated with 15 μM 7rh for the different time points as indicated then stained with CMXRos and MTGreen, and mitochondrial potential was analyzed by flow cytometry. Representative images of flow cytometry (**c**); Results from 3 independent experiments are shown (**d**). Data are shown as the mean ± SD (n = 3) *, *P*< 0.05; **, *P* < 0.01; ***, *P* < 0.001, post hoc comparisons, Tukey's test for results in **a** and **d**.

**Supplementary Figure S3**


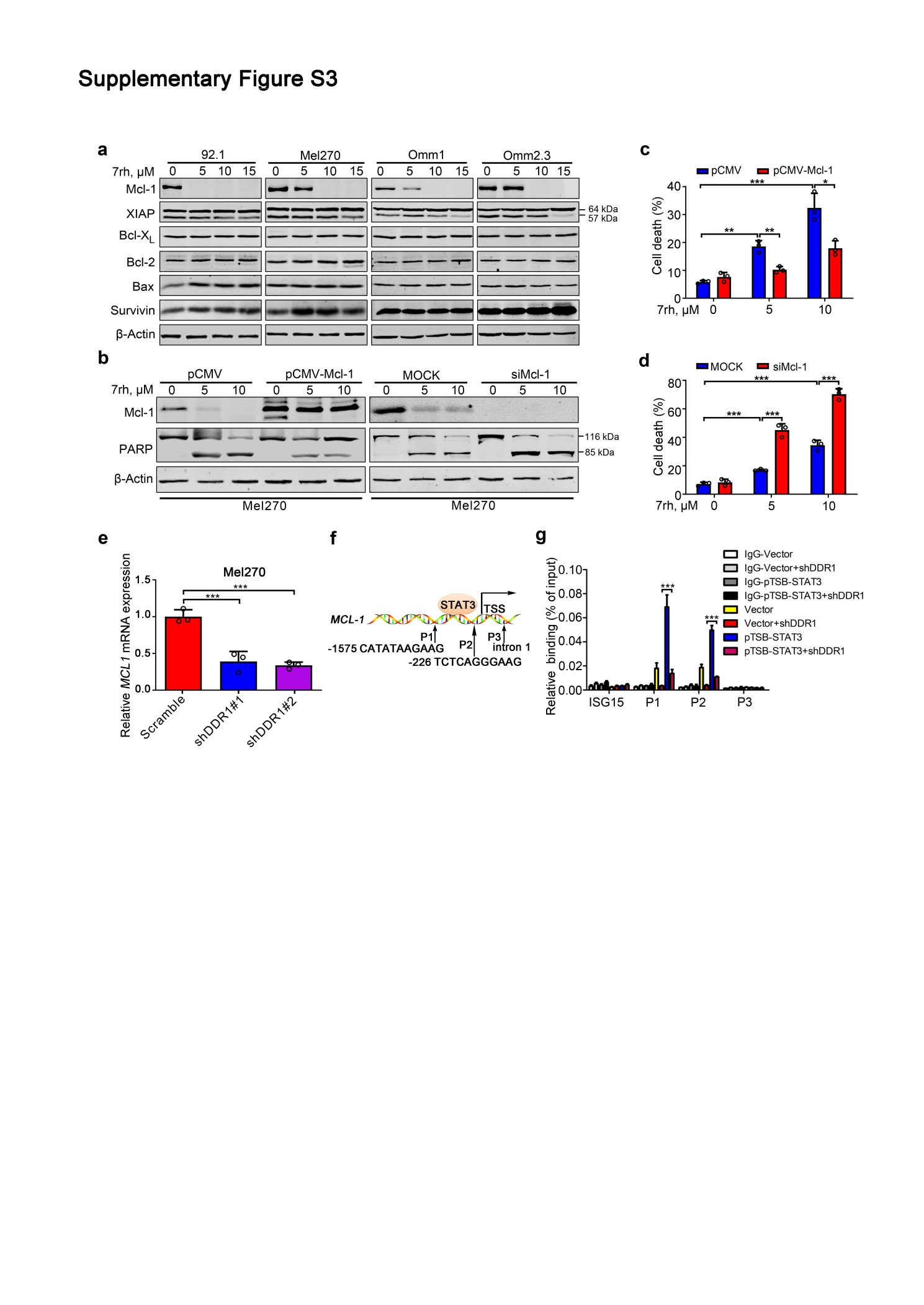


**Supplementary Figure S3. 7rh induces apoptosis by down-regulating STAT3-dependent transcription of Mcl-1 in UM cells. a** The changes in apoptosis-related proteins were determined by Western blotting after 7rh treatment. **b-d** Mel270 cells were transfected with constructs encoding human Mcl-1 or siRNA and then exposed to 7rh for 24 h. Cell death was determined by trypan blue exclusion assay, and whole cell lysates were then analyzed by Western blotting. Data are shown as the mean ± SD (n = 3). **e-g** Mel270 cells transfected with shRNA against DDR1 with or without forced overexpression of STAT3 were subjected to qRT-PCR (**e**) and ChIP assay (**g**). **f** Experimental schematic diagram showing location of STAT3-binding sites of *MCL-1* regulatory region. P1, P2 represent STAT3-binding sites at the *MCL-1* gene promoter. P3 was used as a negative control which located in intronic region. ISG15 as a known non-target gene of STAT3 served as a negative control. **g** ChIP analysis for STAT3 occupancy at the *MCL-1* gene promoter. Data are shown as the mean ± SD (n = 3). *, *P* < 0.05; ***, *P* < 0.001, one-way ANOVA, post hoc comparisons, Tukey's test for results in **e** and **g**; *, *P* < 0.05; ***, *P* < 0.001, Student’s t test for results in **c** and **d**.

**Supplementary Figure S4**


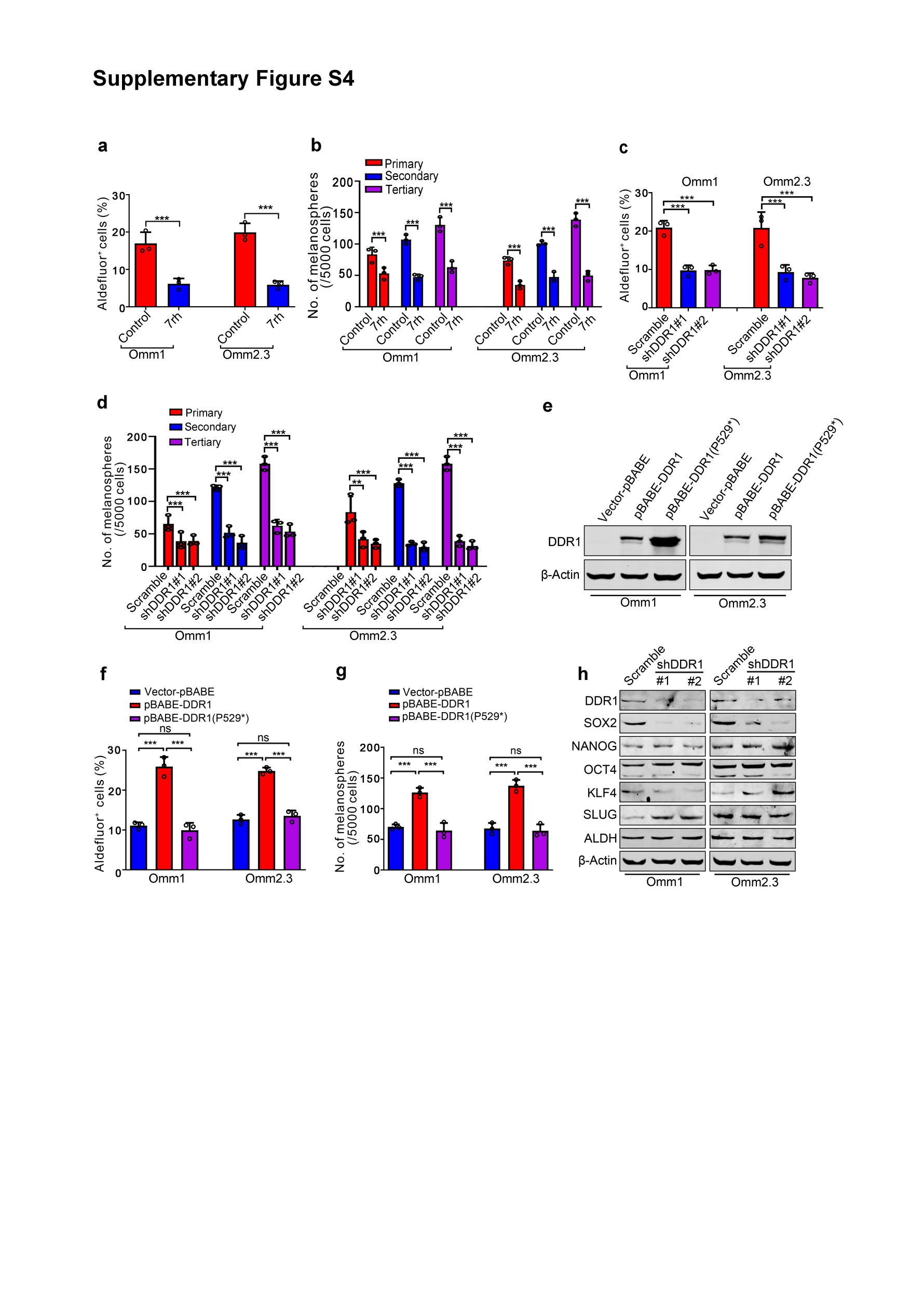


**Supplementary Figure S4. DDR1 promotes CSCs in UM cells. a** Omm1 and Omm2.3 cells were treated with 10 μM 7rh for 48 h, the ALDH activity was detected by flow cytometry. Quantitative analysis of ALDH^+^ cells from 3 independent experiments is shown. Data are shown as the mean ± SD (n = 3). **b** Omm1 and Omm2.3 cells were treated with 10 μM 7rh for 48 h, and then drug-freely cultured for 3 rounds of melanosphere assay for 14 days. Data are shown as the mean ± SD (n = 3). **c** Omm1 and Omm2.3 cells stably transduced with lentiviral Scramble or shDDR1 were assayed by flow cytometry for the proportion of ALDH^+^ cells. Quantitative analysis of ALDH^+^ cells from 3 independent experiments is shown. Data are shown as the mean ± SD (n = 3). **d** Omm1 and Omm2.3 cells stably transduced with lentiviral Scramble or shDDR1 were plated in the stem cell culture medium. Melanosphere units were counted on day 14. The cells were harvested and replated for the secondary and tertiary rounds of evaluation, respectively. Data are shown as the mean ± SD (n = 3). **e** Ectopic expression of wild-type DDR1 or mutant DDR1 (DDR1 P529*) in Omm1 and Omm2.3 cells were determined by Western blotting analysis. **f** Overexpression of wild-type DDR1 but not mutant DDR1 (DDR1 P529*) increased the percentage of ALDH^+^ cells detected by flow cytometry in Omm1 and Omm2.3 cells. ns，no significant. Data are shown as the mean ± SD (n = 3). **g** Ectopic expression of wild-type DDR1 rather than mutant DDR1 (DDR1 P529*) potentiated self-renewal capacity evaluated by melanosphere growth and serially replating assay in Omm1 and Omm2.3. ns, no significant. Data are shown as the mean ± SD (n = 3). **h** DDR1 deletion decreased the expression of SOX2. The protein levels of stemness-related proteins were detected by Western blotting in Omm1 and Omm2.3 cells stably transduced with Scramble or shDDR1 lentivirus. *, *P<* 0.05; ****, *P<* 0.01; *****, *P <* 0.001, Student’s *t* test for results in **a**. *, *P<* 0.05; ***, P<* 0.01; ****, P <* 0.001, one-way ANOVA, post hoc comparisons, Tukey's test for results in **b-d,** and **f-g**.

**Supplementary Figure S5**


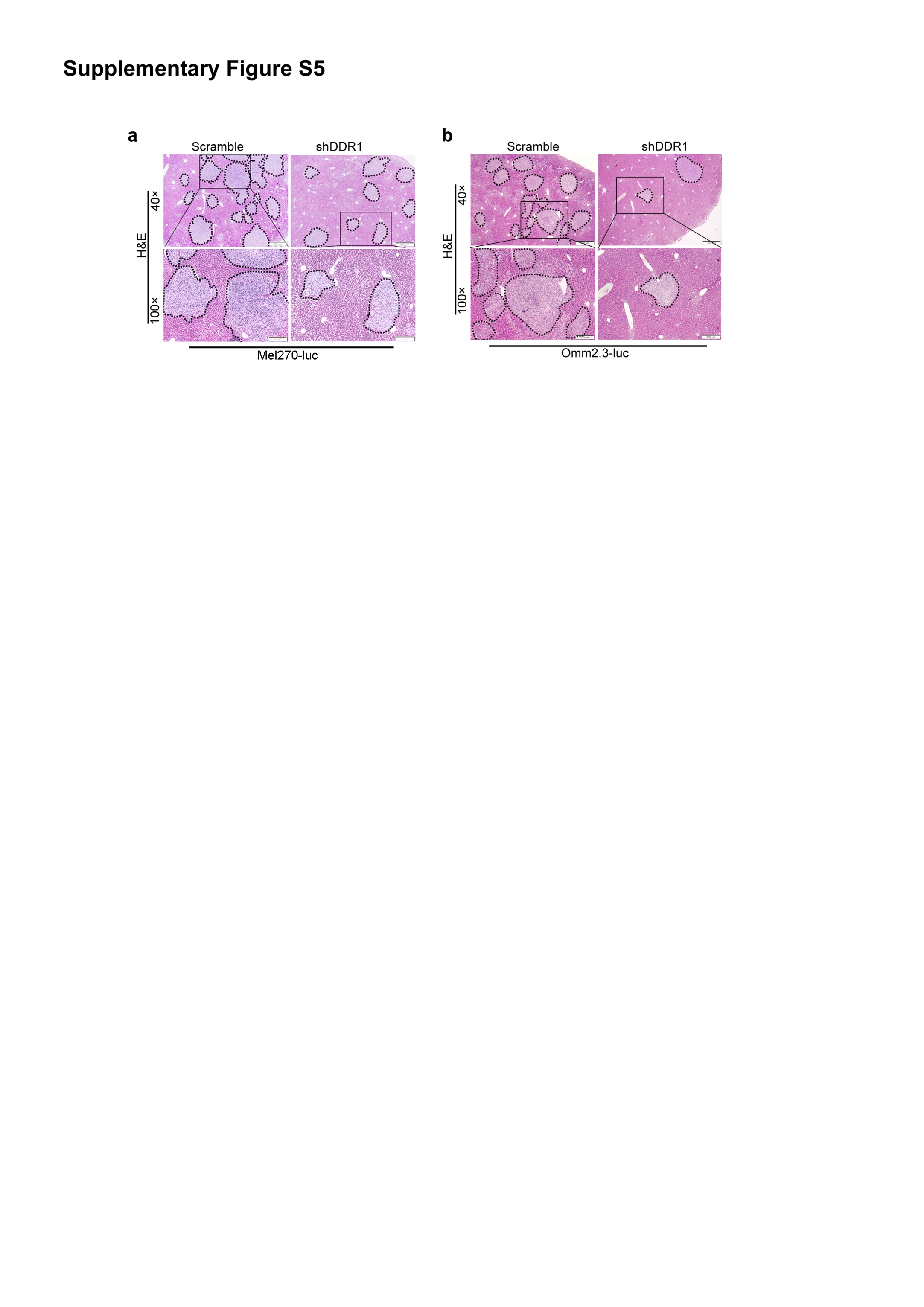


**Supplementary Figure S5. Silencing DDR1 inhibits the metastatic colonization of UM cells in liver. a, b** Metastasis nodules in paraffin sections of liver tissue were identified by H&E staining in Mel270-luc and Omm2.3 cells based metastasis of UM. Scale bar: 500 µm (40×), 200 µm (100×).

**Supplementary Figure S6**


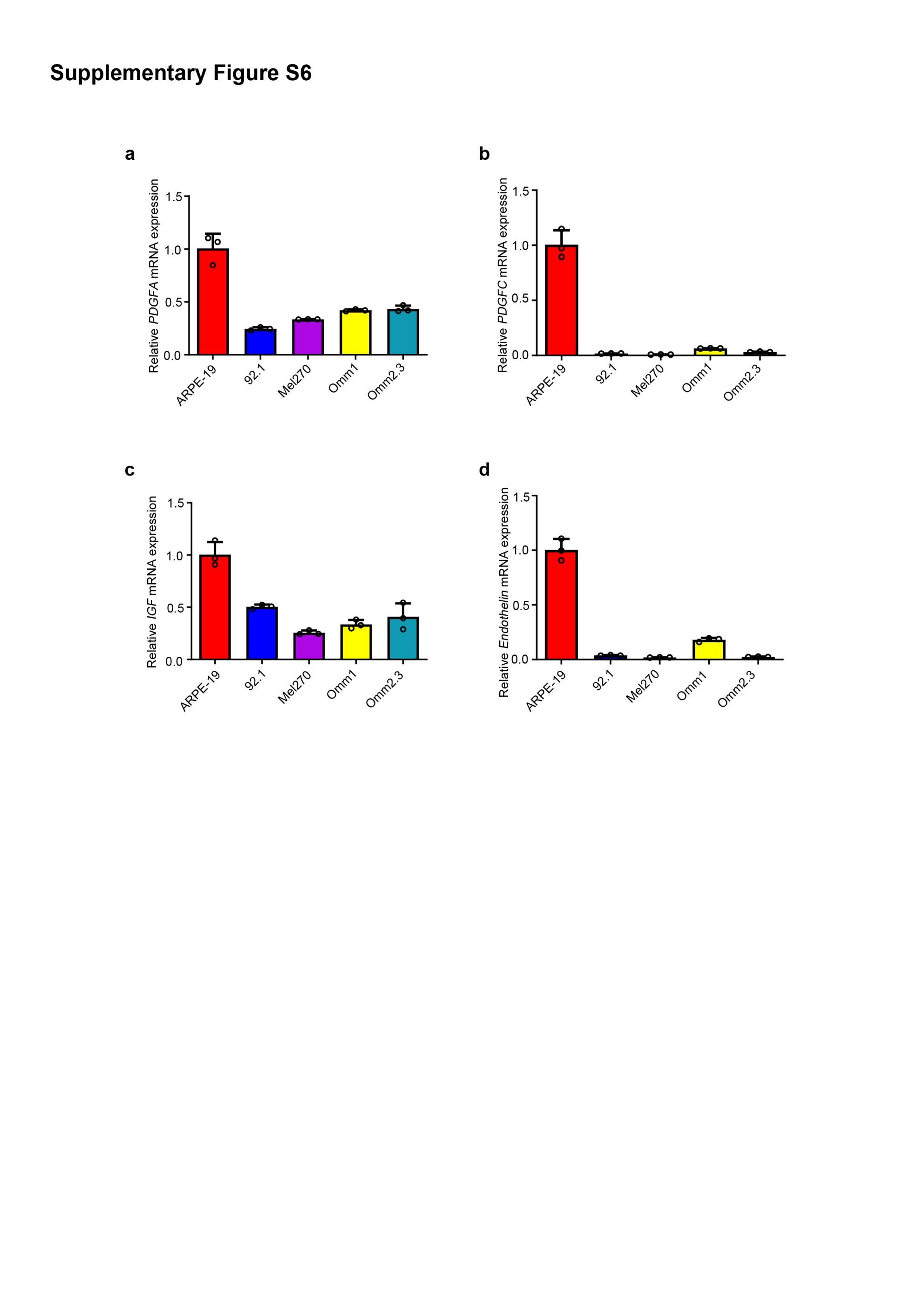


**Supplementary Figure S6. Expression of cytokines in UM cells. a-d** qRT-PCR analysis of PDGFA, PDGFC, IGF, and Endothelin in UM cells and ARPE-19 cells. Data are shown as the mean ± SD (n = 3).

**Supplementary Figure S7**


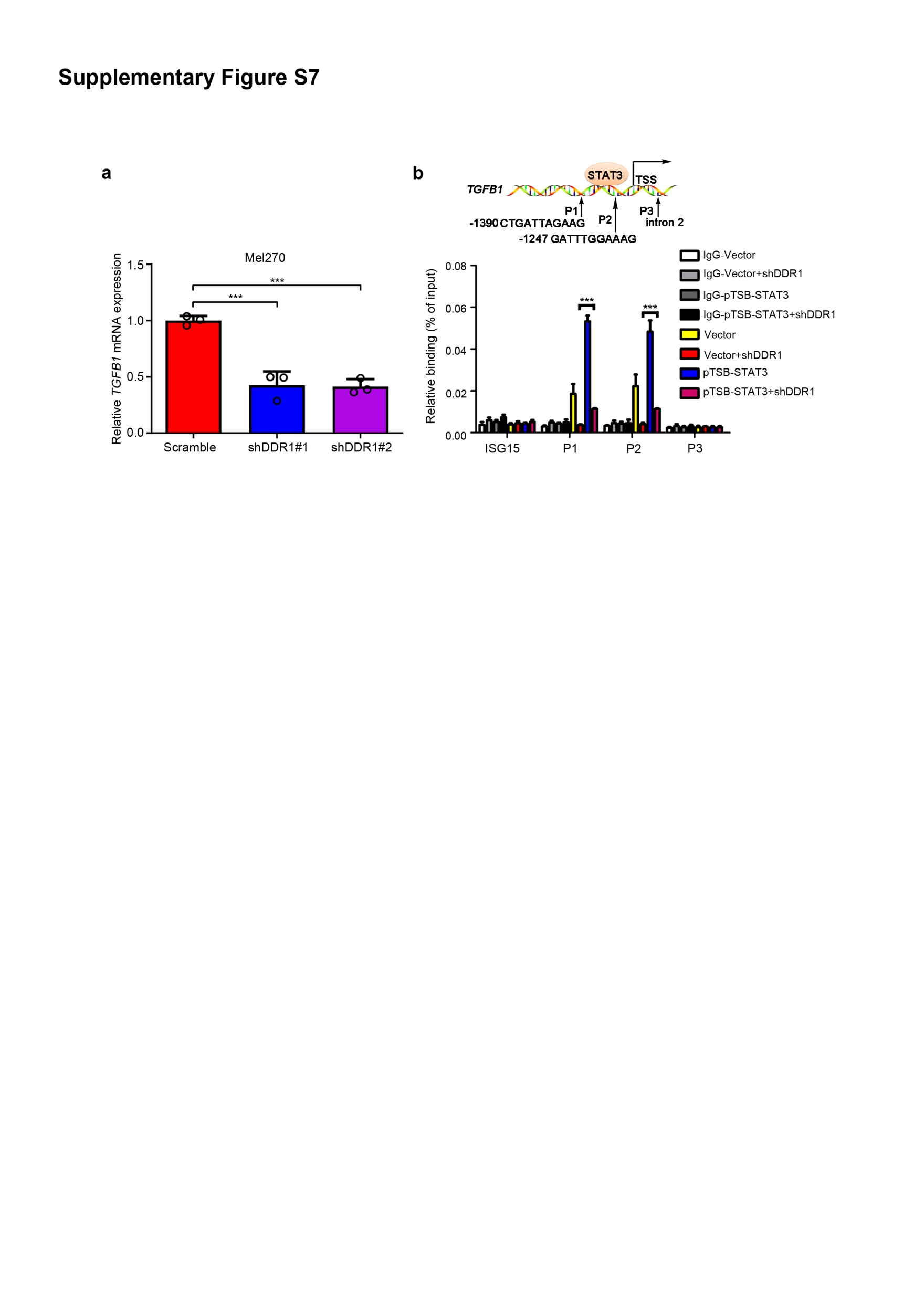


**Supplementary Figure S7. DDR1 increases transcription of TGF-β1 gene in a STAT3-dependent manner. a** qRT-PCR analysis of the mRNA levels of *TGFB1* in Mel270 cells stably expressing Scramble or shRNA against DDR1. Data are shown as the mean ± SD (n = 3). **b** Experimental schematic diagram showing location of STAT3-binding sites of TGF-β1 regulatory region. P1, P2 represent STAT3-binding sites at the *TGFB1* gene promoter. P3 was used as a negative control and is located in intronic region. ISG15 as a known non-target gene of STAT3 served as a negative control. ChIP-PCR analysis for STAT3 occupancy at the *TGFB1* gene promoter in Mel270 cells stably expressed Scramble or shRNA against DDR1. Data are shown as the mean ± SD (n = 3). ***, *P* < 0.001, one-way ANOVA, post hoc comparisons, Tukey's test.

**Supplementary Figure S8**


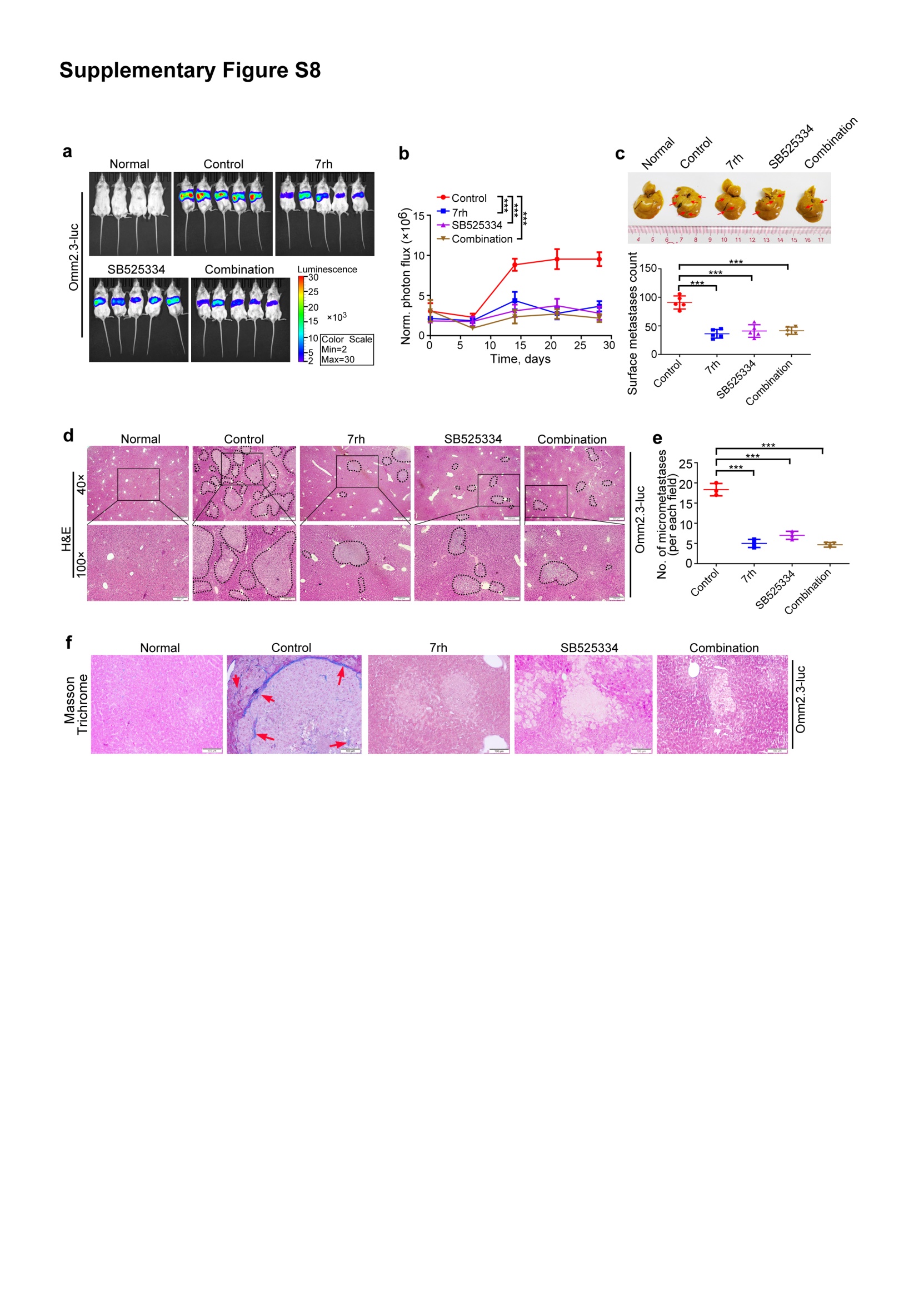


**Supplementary Figure S8. 7rh or SB525334 abrogates liver metastatic colonization by interrupting the TGF-β1-collagen I-DDR1-STAT3 loop in UM.**

After 5×10^5^ Omm2.3-luc cells were intrasplenically inoculated, the NOG mice were administrated with vehicle (ddH_2_O:DMSO:EtOH:Cremophor EL=90:2:4:4), 7rh (25 mg/kg, orally), SB525334 (30 mg/kg/day, i.p.) alone or combination 7rh (25 mg/kg, orally) with SB525334 (30 mg/kg/day, i.p.) every day for 28 days (n=5 per group). **a** Representative images of luciferase signals on day 28 after treatment with vehicle, 7rh, SB525334 alone or combination 7rh with SB525334. **b** quantitative analysis of photon flux for hepatic metastases in NOG mice was performed every week. Data are shown as the mean ± SD (n = 5). **c** The mice were sacrificed to count metastatic nodules on liver surface. Representative images and quantitative analysis of liver nodules are shown. Data are shown as the mean ± SD (n = 5). **d, e** Metastasis nodules in paraffin sections of liver tissue from each group were identified by H&E staining. Representative images and quantitative analysis of nodules are shown. Data are shown as the mean ± SD (n = 3). Scale bar: 500 µm (40×), 200 µm (100×). **f** Collagens were detected by Masson trichrome staining in paraffin sections of liver tissue. Scale bar: 200 µm (100×). ***, *P* < 0.001, Student’s *t* test for results in **b**. *****, *P <* 0.001, one-way ANOVA, post hoc comparisons, Tukey's test for results in **c** and **e**.

**Supplementary Figure S9**


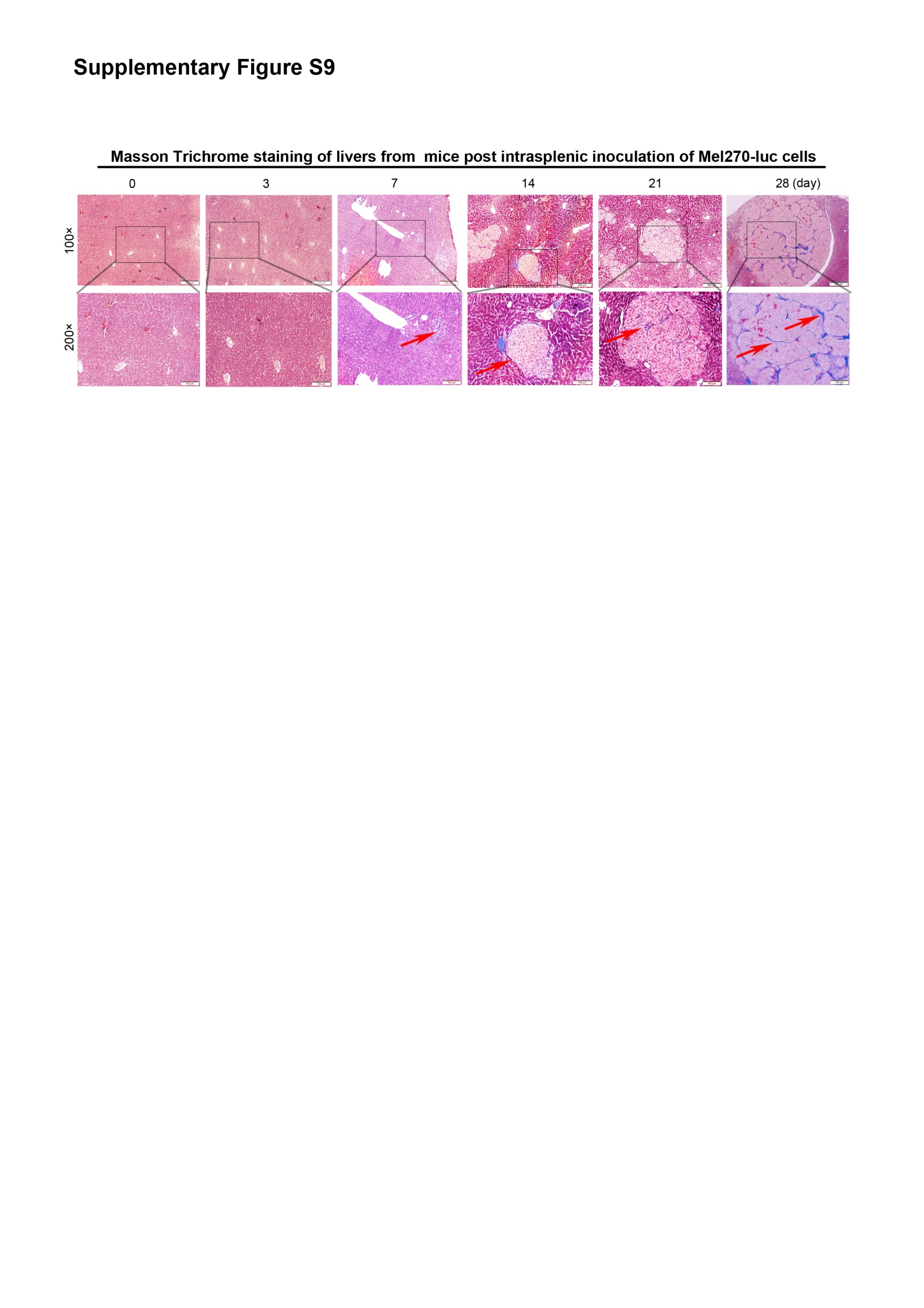


**Supplementary Figure S9. Masson Trichrome staining of liver sections from mice post intrasplenic inoculation of Mel270-luc cells.** Blue: Collagen staining. Red arrows indicate collagen surrounding the liver tumor nodules. Scale bar: 200 µm (100×), 100 µm (200×).

**Supplementary Figure S10**


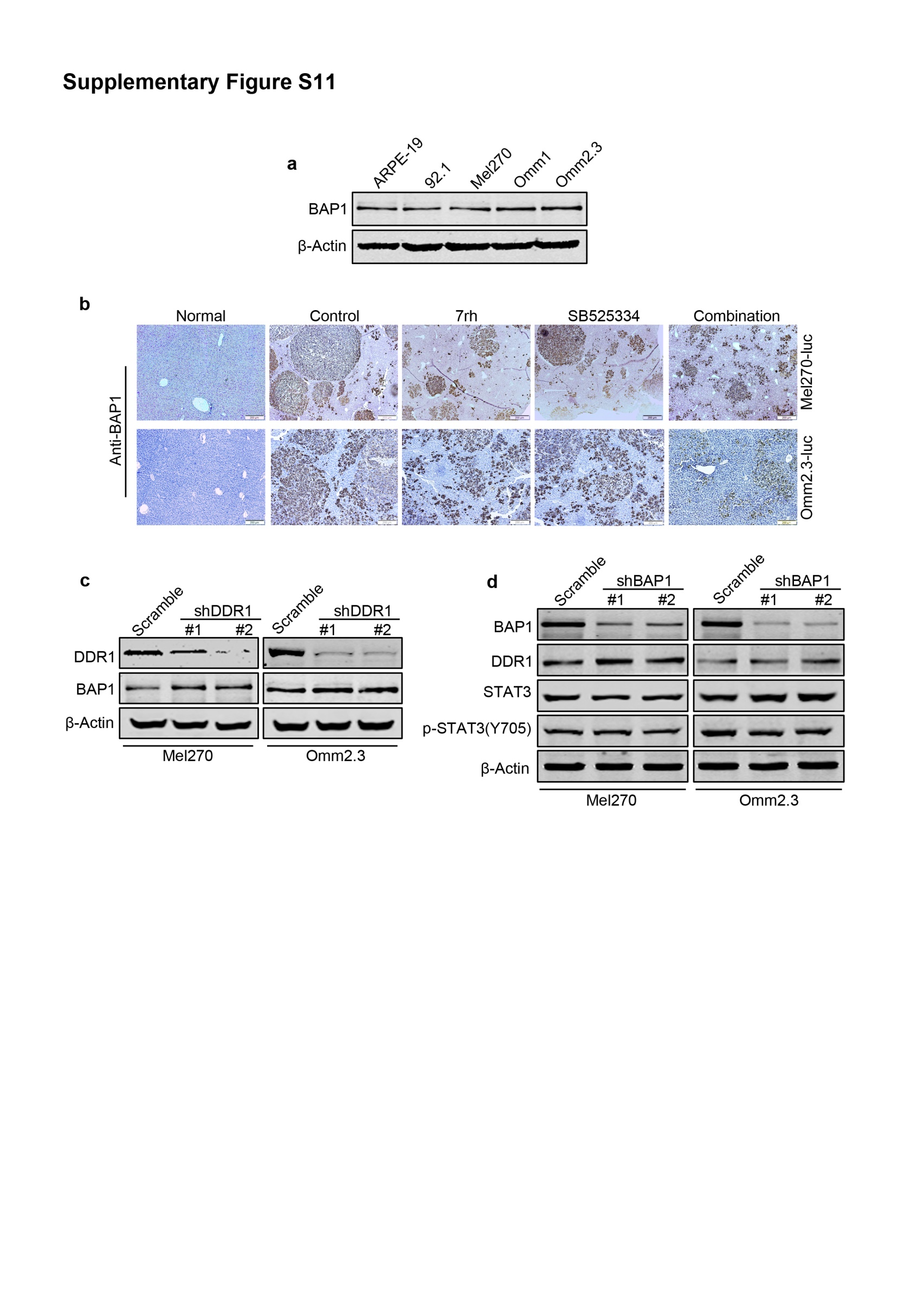


**Supplementary Figure S10. The expression of BAP1 and DDR1 is independent in UM cells.**

**a** Protein levels of BAP1 in ARPE-19 cells and four UM cell lines (92.1, Mel270, Omm1, Omm2.3) were determined by Western blotting analysis. **b** IHC staining of BAP1 in sections of liver metastasis form animal studies. **c** Mel270 and Omm2.3 cells were transfected with Scramble (pLKO.1-puro-Non-target shRNA) or human DDR1 specific target shRNA (pLKO.1-puro-DDR1-target shRNA), the expression of BAP1 was evaluated by Western blotting analysis. **d,** Mel270 and Omm2.3 cells were stably knocked down BAP1, Western blotting analysis was employed to determine DDR1 and its downstream signal phospho-STAT3.

**Supplementary Figure S11**


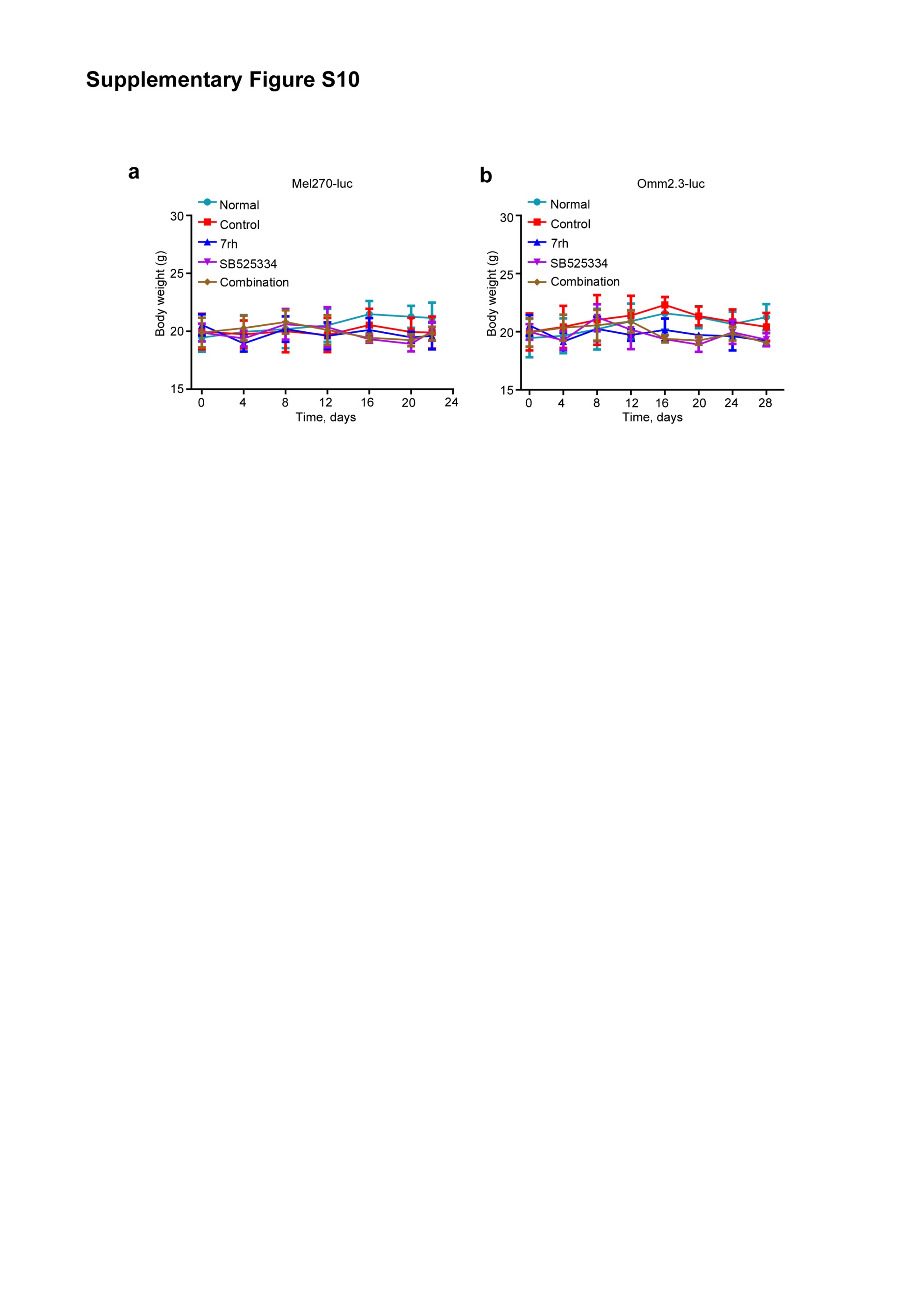


**Supplementary Figure S11. Treatment with 7rh or SB525334 exhibited negligible toxicity to the**

**mice as reflected by body weight changes. a, b** NOG mice were intrasplenically injected with

Mel270-luc or Omm2.3-luc cells and administered with vehicle, 7rh (25 mg/kg, orally) or SB525334

(30 mg/kg/day, i.p.) for 22 days (Mel270-luc cells) or 28 days (Omm2.3-luc cells), body weight of

mice was measured every 4 days and quantitative analysis of the data was shown. Data are shown as the mean ± SD (n = 5).

**Supplementary Table S1. Detailed information for 62 patients with UM**

| Pt# | S/Y | Location | LBD (mm) | Thickness (mm) | | Therapy | | Vimentin | | HMB45 | | S100 | | Ki67 | | TNM | | DDR1 | |
| --- | --- | --- | --- | --- | --- | --- | --- | --- | --- | --- | --- | --- | --- | --- | --- | --- | --- | --- | --- |
| 1 | F/37 | Choroid | 13.0 | 13.0 | | Enucleation | | + | | + | | + | | 20% | | T3 | | M | |
| 2 | M/27 | Choroid | 6.1 | 8.1 | | Enucleation | | + | | + | | + | | 10% | | T2 | | M | |
| 3 | F/47 | Choroid | 8.8 | 12.8 | | Enucleation | | + | | + | | + | | 15% | | NA | | L | |
| 4 | M/64 | Choroid | 9.0 | 9.0 | | Enucleation | | + | | + | | + | | 10% | | T2 | | L | |
| 5 | M/56 | Choroid | 9.9 | 12.2 | | Enucleation | | + | | + | | + | | 10% | | T3 | | M | |
| 6 | M/47 | Choroid | 10.2 | 9.2 | | Enucleation | | + | | + | | + | | 10% | | T3 | | H | |
| 7 | F/51 | Choroid | 12.8 | 5.0 | | Enucleation | | + | | + | | + | | 10% | | T2 | | M | |
| 8 | F/45 | Choroid | 10.6 | 8.2 | | Enucleation | | + | | + | | + | | 20% | | T2 | | N | |
| 9 | F/63 | Choroid, | 10.7 | 9.5 | | Enucleation | | + | | + | | + | | 8% | | T3 | | M | |
| 10 | M/48 | Choroid | 11.0 | 8.5 | | Enucleation | | + | | + | | + | | 10% | | T2 | | H | |
| 11 | F/64 | Choroid | 11.1 | 15.9 | | Enucleation | | + | | + | | + | | 25% | | T4 | | M | |
| 12 | M/37 | Choroid | 11.2 | 10.1 | | Enucleation | | + | | + | | + | | 20% | | T3 | | M | |
| 13 | M/44 | Choroid | 11.3 | 9.0 | | Enucleation | | + | | + | | + | | 20% | | T3 | | M | |
| 14 | F/57 | Choroid | 9.9 | 11.3 | | Enucleation | | + | | + | | + | | 15% | | T3 | | M | |
| 15 | M/49 | Choroid | 11.4 | 10.4 | | Enucleation | | + | | + | | + | | 20% | | T3 | | M | |
| 16 | F/46 | Choroid sclera ingrowth | 11.5 | 8.3 | | Enucleation | | + | | + | | + | | 15% | | T2 | | N | |
| 17 | M/60 | Choroid | 11.5 | 6.4 | | Enucleation | | + | | + | | + | | 15% | | T2 | | M | |
| 18 | F/45 | Choroid | 11.5 | 10.1 | | Enucleation | | + | | + | | + | | <5% | | T3 | | M | |
| 19 | F/60 | Choroid | 11.5 | 9.4 | | Enucleation | | + | | + | | + | | 10% | | T3 | | M | |
| 20 | M/44 | Choroid | 11.6 | 4.1 | | Enucleation | | + | | + | | + | | 10% | | T2 | | M | |
| 21 | M/41 | Choroid/Ciliary body | 11.8 | 11.3 | | Enucleation | | + | | + | | + | | <5% | | T3 | | M | |
| 22 | M/64 | Choroid | 11.8 | 6.2 | | Enucleation | | + | | + | | + | | 10% | | T2 | | M | |
| 23 | M/61 | Choroid | 11.8 | 8.6 | | Enucleation | | + | | + | | + | | 20% | | T2 | | M | |
| 24 | F/60 | Choroid | 11.8 | 11.4 | | Enucleation | | + | | + | | + | | 10% | | T3 | | M | |
| 25 | M/64 | Choroid | 12.0 | 11.0 | | Enucleation | | + | | + | | + | | <5% | | T3 | | M | |
| 26 | F/41 | Choroid  Ciliary body | 12.2 | 10.3 | | Enucleation | | + | | + | | + | | 10% | | T3 | | M | |
| 27 | M/61 | Choroid | 12.5 | 8.4 | | Enucleation | | + | | + | | + | | 30% | | T3 | | H | |
| 28 | F/50 | Choroid | 12.5 | | 11.2 | | Enucleation | | + | | + | | + | | <5% | | T3 | | M |
| 29 | M/60 | Choroid | 12.5 | | 9.3 | | Enucleation | | + | | + | | + | | 10% | | T3 | | M |
| 30 | F/57 | Choroid, | 12.5 | | 6.8 | | Enucleation | | + | | + | | + | | 20% | | T3 | | M |
| 31 | M/38 | Choroid | 12.8 | 6.6 | | Enucleation | | + | | + | | + | | 10% | | | T3 | M | |
| 32 | F/48 | Choroid | 13.0 | 9.3 | | Enucleation | | + | | + | | + | | 10% | | | T3 | M | |
| 33 | F/45 | Choroid | 13.3 | 11.8 | | Enucleation | | + | | + | | + | | 10% | | | T3 | H | |
| 34 | F/49 | Choroid  sclera ingrowth | 13.3 | 7.5 | | Enucleation | | + | | + | | + | | 10% | | | T3 | N | |
| 35 | M/28 | Choroid | 13.5 | 10.5 | | Enucleation | | + | | + | | + | | 5% | | | T3 | M | |
| 36 | M/51 | Choroid | 13.9 | 16.6 | | Enucleation | | + | | + | | + | | 10% | | | T4 | H | |
| 37 | M/49 | Choroid  sclera ingrowth | 14.1 | 13.4 | | Enucleation | | + | | + | | + | | 5% | | | T3 | N | |
| 38 | F/45 | Choroid | 14.1 | 9.4 | | Enucleation | | + | | + | | + | | 20% | | | T3 | H | |
| 39 | M/41 | Choroid | 14.2 | 8.2 | | Enucleation | | + | | + | | + | | 20% | | | T3 | H | |
| 40 | M/67 | Choroid | 14.3 | 11.4 | | Enucleation | | + | | + | | + | | 11% | | | T3 | H | |
| 41 | F/51 | Choroid | 14.5 | 11.6 | | Enucleation | | + | | + | | + | | 15% | | | T3 | H | |
| 42 | M/71 | Choroid | 14.7 | 14.2 | | Enucleation | | + | | + | | + | | 15% | | | T3 | H | |
| 43 | M/73 | Choroid | 14.9 | 13.4 | | Enucleation | | + | | + | | + | | 20 | | | T3 | H | |
| 44 | M/44 | Choroid | 15.1 | 14.6 | | Enucleation | | + | | + | | + | | 20% | | | T4 | N | |
| 45 | M/63 | Choroid | 15.4 | 11.9 | | Enucleation | | + | | + | | + | | 40% | | | T3 | H | |
| 46 | M/60 | Choroid | 15.4 | 17.8 | | Enucleation | | + | | + | | + | | 20% | | | T4 | H | |
| 47 | F/50 | Choroid | 15.6 | 12 | | Enucleation | | + | | + | | + | | 10% | | | T3 | H | |
| 48 | F/46 | Choroid | 15.9 | 9.0 | | Enucleation | | + | | + | | + | | 25% | | | T3 | M | |
| 49 | M/62 | Choroid/Ciliary body | 16.3 | 9.7 | | Enucleation | | + | | + | | + | | <5% | | | T3 | M | |
| 50 | M/43 | Choroid | 16.3 | 10.4 | | Enucleation | | + | | + | | + | | <5% | | | T3 | M | |
| 51 | M/30 | Choroid | 16.4 | 11.0 | | Enucleation | | + | | + | | + | | <5% | | | T3 | H | |
| 52 | M/52 | Choroid | 16.4 | 12.9 | | Enucleation | | + | | + | | + | | 10% | | | T4 | H | |
| 53 | F/60 | Choroid/Ciliary body | 16.5 | 10.7 | | Enucleation | | + | | + | | + | | 15% | | | T3 | M | |
| 54 | M/77 | Choroid | 16.9 | 15.9 | | Enucleation | | + | | + | | + | | 5% | | | T4 | H | |
| 55 | F/35 | Choroid | 17.0 | 15.0 | | Enucleation | | + | | + | | + | | 10% | | | T4 | H | |
| 56 | F/52 | Choroid/Ciliary body | 17.4 | 13.0 | | Enucleation | | + | | + | | + | | 10% | | | T4 | M | |
| 57 | M/42 | Choroid | 18.3 | 17.1 | | Enucleation | | + | | + | | + | | 10% | | | T4 | H | |
| 58 | F/45 | Choroid | 18.9 | 7.9 | | Enucleation | | + | | + | | + | | 50% | | | T4 | M | |
| 59 | M/67 | Choroid | 19.3 | 10.5 | | Enucleation | | + | | + | | + | | 15% | | | T4 | M | |
| 60 | M/65 | Choroid | 21.2 | 12.1 | | Enucleation | | + | | + | | + | | 30% | | | T4 | H | |
| 61 | M/61 | Choroid | 22.1 | 10.6 | | Enucleation | | + | | + | | + | | 10% | | | T4 | H | |
| 62 | M/41 | Choroid | 23.2 | 21.3 | | Enucleation | | + | | + | | + | | 30% | | | T4 | H | |

Pt#: Patient number; S/Y: Sex/Year; LBD: Largest basal diameter; N: Negative; L: Low; M: Medium; H: High; TNM: T, Primary tumor; N, Regional lymph node metastasis; M, Systemic metastasis. NA: not available

**Supplementary Table S2. The association between DDR1 expression and clinicopathologic features in UM patients**

| **Variables** | **DDR1** | | | | |  |
| --- | --- | --- | --- | --- | --- | --- |
|  | **Cases** | **Negative** | **Low** | **Medium** | **High** | **p Value§** |
| Age (y) |  |  |  |  |  | 0.1624 |
| ≤50  >50 | 32  30 | 5  0 | 1  1 | 16  17 | 10  12 |  |
| Gender  Male  Female | 37  25 | 2  3 | 1  1 | 17  16 | 17  5 | 0.0661 |
| The largest basal diameter (mm)  ≤15  >15 | 43  19 | 4  1 | 2  0 | 26  7 | 11  11 | 0.00006 |
| Thickness (mm)  ≤12  >12 | 44  18 | 2  3 | 1  1 | 29  4 | 12  10 | 0.0386 |
| Ki67 expression level  ≤10%  >10% | 34  28 | 2  3 | 1  1 | 20  13 | 11  11 | 0.3948 |
| TNM Stage  T1  T2  T3  T4 | 0  10  37  14 | 0  2  2  1 | 0  1  0  0 | 0  6  23  4 | 0  1  12  9 | 0.0019 |

§ Chi-square test was independently applied for each variable. The largest basal diameter (15 mm) and Thickness (12 mm) are medians of the cohort.

**Supplementary Table S3. DDR1 deletion down-regulates the *in vivo*** **frequency of CSCs in UM cells**

**Limiting dilution assays**

|  | **Engrafted mice** |  |
| --- | --- | --- |
| Cell number  3×10^6^  1×10^6^  5×10^5^  1×10^5^  Frequency | Scramble  6/6  5/6  0/6  0/6  1/1,095,854 | shDDR1  2/6  2/6  0/6  0/6  1/5,828,802 |

**Supplementary Table S4. Primers for qRT-PCR analysis**

| **Genes** | **Forward primers** | **Reverse primers** |
| --- | --- | --- |
| *DDR1*  *DDR2*  *MCL1*  *SOX2*  *TGFB1*  *COL1A1*  *GAPDH* | 5'-CCCCAATGGCTCTGCCTA-3'  5'-TGTTCCTGCTGCCTATCTT-3'  5'-GCATGCTTCGGAAACTGGAC-3'  5'-CAGAAAAACAGCCCGGACC-3'  5'-TGGTGGAAACCCACAACGAA-3'  5'-GGCGGCCAGGGCTCCGACCC-3'  5'-GATCGAATTAAACCTTATCGTCGT-3' | 5'-AACAATGTCAGCCTCGGCATA-3'  5'-AGGATAGCGGCATATAGCTGGAT-3'  5'-GAGAGTCACAATCCTGCCCC-3'  5'-TTGCTGATCTCCGAGTTGTG-3'  5'-GAGCAACACGGGTTCAGGTA-3'  5'-AATTCCTCGTCTGGGGCACC-3'  5'-AGCAGCAGAACTTCCACTCGGT-3' |

**Supplementary Table S5. Primers for ChIP assay**

|  | **Primers** | **Sequences** |
| --- | --- | --- |
| P1  P2  P3  P1  P2  P3  P1  P2  P3 | *MCL1* promoter forward  *MCL1* promoter reverse  *MCL1* promoter forward  *MCL1* promoter reverse  *MCL1* promoter forward  *MCL1* promoter reverse  *SOX2* promoter forward  *SOX2* promoter reverse  *SOX2* promoter forward  *SOX2* promoter reverse  *SOX2* promoter forward  *SOX2* promoter reverse  *TGFB1* promoter forward  *TGFB1* promoter reverse  *TGFB1* promoter forward  *TGFB1* promoter reverse  *TGFB1* promoter forward  *TGFB1* promoter reverse  *ISG15* promoter forward  *ISG15* promoter reverse | 5'-AGATCTTTTAACAAAAAGGCTGCT-3'  5'-GCCCTTGAGAACAAGGATCTTC-3'  5'-CTCTGGCGAAAACCAGCAAA-3'  5'-TCTTTCCATGAGCACCTCGG-3'  5'-GCGGTAATCGGACTCAACCT-3'  5'-TGGCCAAAAGTCGCCCTC-3'  5'-AAAAGGAGAACCTTCGGGGG-3'  5'-ACCCACTGGAAACTCAGAAAAG-3'  5'-ACCCATTTATTCCCTGACAGC-3'  5'-TTGCTTTCTTGGCTGAGCAC-3'  5'-AAAAACGAGGGAAATGGGAGG-3'  5'-AGCGTACCGGGTTTTCTCC-3'  5'-ATGGGGTCATGGAGGAGGATAA-3'  5'-TCCTTCCATAGCTCCCCAGTG-3'  5'-GAAAGGAGGCTGGGTTGGAA-3'  5'-GACTCCTGCTGATTCCCCAC-3'  5'-CTGTGGCTACTGGTGCTGAC-3'  5'-GCAGCTTGGACAGGATCTGG-3'  5'-GATCACCCAGAAGATCGGC-3'  5'-GCCCTTGTTATTCCTCACCA-3' |
